# Adaptive kernel fuzzy clustering for missing data

**DOI:** 10.1371/journal.pone.0259266

**Published:** 2021-11-12

**Authors:** Anny K. G. Rodrigues, Raydonal Ospina, Marcelo R. P. Ferreira

**Affiliations:** 1 Departamento de Estatística, CASTLab, CCEN, Universidade Federal de Pernambuco, Cidade Universitária, Recife, PE, Brazil; 2 Departamento de Estatística, DataLab, Centro de Ciências Exatas e da Natureza, Universidade Federal da Paraíba, João Pessoa, PB, Brazil; University of Technology Malaysia: Universiti Teknologi Malaysia, MALAYSIA

## Abstract

Many machine learning procedures, including clustering analysis are often affected by missing values. This work aims to propose and evaluate a Kernel Fuzzy C-means clustering algorithm considering the kernelization of the metric with local adaptive distances (VKFCM-K-LP) under three types of strategies to deal with missing data. The first strategy, called Whole Data Strategy (WDS), performs clustering only on the complete part of the dataset, i.e. it discards all instances with missing data. The second approach uses the Partial Distance Strategy (PDS), in which partial distances are computed among all available resources and then re-scaled by the reciprocal of the proportion of observed values. The third technique, called Optimal Completion Strategy (OCS), computes missing values iteratively as auxiliary variables in the optimization of a suitable objective function. The clustering results were evaluated according to different metrics. The best performance of the clustering algorithm was achieved under the PDS and OCS strategies. Under the OCS approach, new datasets were derive and the missing values were estimated dynamically in the optimization process. The results of clustering under the OCS strategy also presented a superior performance when compared to the resulting clusters obtained by applying the VKFCM-K-LP algorithm on a version where missing values are previously imputed by the mean or the median of the observed values.

## 1 Introduction

The incessant increase in volume and variety of data requires advances in methodologies in order to understand, process and summarize data automatically. Cluster analysis is one of the main unsupervised techniques that are used to extract knowledge from data, due to its ability to aid in the process of understanding and visualizing data structures [1, 2].

The main goal in clustering is to organize the data (observations, data items, images, pixels etc.) based on similarity (or dissimilarity) criteria such that observations belonging to the same group show high degrees of similarity, while observations in different groups show high degrees of dissimilarity [3, 4].

Clustering methods are widely used in many areas of knowledge, such as taxonomy, data mining, image segmentation, pattern recognition, information retrieval, computer vision, and so forth [3, 5]. Depending on the application considered, the groups obtained in clustering may present different characteristics. Thus, different clustering techniques have been proposed in the literature, with the most popular ones being based on hierarchies and partitions. In hierarchical clustering algorithms, structures are found such that they can be recursively divided into levels. The output is a nested sequence of partitions of the input data known as a dendrogram [6].

In partitioning clustering methods, a single partition of the dataset is obtained, generally based on the optimization of a suitable objective function [5]. These methods are more flexible than the hierarchical ones because they allow observations to change groups at each step of the algorithm, if that change leads to a better solution in terms of the variability of the resulting partition. Partitioning clustering methods can be divided into two main branches: hard (or crisp) and fuzzy (or soft). In hard clustering methods, the groups are naturally disjoint, that is, the dataset is partitioned into a predefined number of groups and overlapping is not allowed, which means that each instance may belong exactly to one cluster.

In real world applications, group boundaries are often difficult to define, as it is complex to find reasonable criteria that include some data objects in a cluster, but exclude others. Trying to solve this problem, methods that allow more flexible criteria, such as fuzzy clustering algorithms, were proposed in the literature. In fuzzy clustering, an instance may belong simultaneously to all clusters with a certain membership degree [7, 8]. Fuzzy clustering methods offer good capability to handle noisy/missing data, which is a common problem in different areas, including microarray data analysis [3, 4, 9–11].

The most important component of any clustering algorithm is the dissimilarity (or similarity) measure. Distances are important examples of dissimilarity measures and the Euclidean distance is the most commonly used in the clustering literature. The Fuzzy *C*-Means (FCM) method [12] is one of the most popular clustering algorithms and it is based on the Euclidean distance. Algorithms that are based on this distance achieve good results when applied to datasets in which groups are approximately hyperspherical and approximately linearly separable [13]. In the opposite situation, i.e. clusters with non-hyperspherical shapes and/or linearly non-separable patterns), these algorithms may have poor performance and find unrepresentative clusters.

The seminal work by Girolami [14] introduces the kernel *K*-means algorithm that generalizes several clustering methods [15] that produce hypersurfaces with nonlinear separation between groups, such as the Kernel Fuzzy *C*-Means [5, 16, 17], Kernel-based Self-Organizing Maps (SOM) [18, 19], Kernel Neural Gas [20] and Kernel Subtractive Clustering [21, 22]. Several studies have shown the superiority of the kernel-based clustering methods in a variety of real-world problems [23–27].

The use of kernel functions allows an arbitrary nonlinear mapping *ϕ* from the original *p*-dimensional space of the dataset $X\subset R^{p}$ to a higher-dimensional (possibly infinite) space, called a feature space F. The purpose of this transformation is that by moving to higher dimensions it may be possible to obtain more defined and linearly separable groups [28]. The advantage and, at the same time, the main idea of methods based on kernel functions is that inner products in the feature space can be expressed as a Mercer kernel [14, 29]. Two main approaches have guided the development of kernel-based algorithms: kernelization of the metric, in which the cluster prototypes are obtained in the original space and the distances between instances and cluster prototypes are computed by means of kernels; and clustering in feature space, in which cluster prototypes are obtained in the feature space [17].

Research studies have shown that clustering methods based on kernel functions perform better than traditional methods, as they are able to produce nonlinear differentiable hypersurfaces of separation between groups [5, 17]. However, in most domains, especially if we are dealing with high-dimensional datasets, some variables may be irrelevant for the construction of the groups, and some among the relevant may be less important than others in relation to a specific group. Ferreira et al. [13] proposed a family of methods based on kernel functions with automatic weighting of variables. These methods were derived based on kernelized adaptive distances that change at each algorithm iteration and can be different for each group or common to all groups. In this context, the Kernel Fuzzy *C*-Means clustering under the kernelization of the metric approach with local adaptive distances was considered, assuming the constraint that the product of the weights of the variables on each cluster must be equal to one. In this work, we labeled this algorithm as VKFCM-K-LP.

Ferreira, et al. [13] focused on developing methods that are able to better describe the structures of groups in data, however, they did not investigate the performances of the algorithms in the context of missing data. In real world applications, many inferential procedures have to deal with the problem of missing data. There are several reasons for this problem, including imperfect manual data entry procedures, incorrect measurement and equipment measurement errors, among others [30].

In many areas, such as Industry and Medicine, it is common to find datasets that have up to 50% or more of missing values [31, 32]. Extensive research has been done to study the problem of missing data, and the reason for this is the fact that many statistics were originally developed for datasets with no missing values, and even a small amount of them in the dataset can cause serious problems in analysis and decision making. This is enough to motivate the need to develop efficient mechanisms to deal with incomplete data [33].

The development of statistical methods to deal with incomplete data has been the subject of research for decades [34–36]. Green et al. [37] assessed two alternatives for dealing with missing values: Imputation, in which the missing values are estimated through the values observed in the dataset, of which the most popular techniques are Average Imputation or Median Imputation; and Exclusion, where observations that contain missing values are excluded from the dataset. Although simple, these alternatives can produce biased estimates through the reduction of the size of the dataset and by replacing these missing values with estimates [35]. A more effective approach can be to adapt traditional data analysis to deal with incomplete data.

Several approaches have been introduced in an attempt to extend the clustering techniques in the presence of missing values. One of the first attempts was an approach based on probabilistic assumptions to handle missing data in order to perform pattern recognition [38] introduces an approach based on probabilistic assumptions to handle missing data. The Expectation-Maximization (EM) algorithm was used to deal with incomplete data in clustering [39]. Several methods have been proposed to adapt the FCM method to deal with missing data [40]. Wagstaff [41] proposed the *K*-means method with Soft Constraints (KSC) and Poddar et al. [42] examine clustering data with missing entries using non-convex fusion penalties.

Hathaway [43] proposed strategies to deal with missing values in cluster analysis using the FCM method. Li et al. [44, 45] proposed the FCM clustering method based on nearest-neighbor observations and extended the FCM method by adding a variable weighting process to handle incomplete data, in which the weight of each attribute is seen as an additional variable to be optimized simultaneously in clustering. Recently, Li et al. [46] introduced a kernel method to cluster datasets with missing values in the scope of imputation of observations.

In this work, we adapted the VKFCM-K-LP clustering methods [13, 43] to deal with missing data. The first strategy, called Whole Data Strategy (WDS) performs clustering only on the complete part of the dataset, which means that, in this first strategy, the instances that contain any missing value are excluded from the analysis. The WDS can be applied as long as the amount of missing values does not exceed a percentage of 25% of all observed values. The second approach uses the Partial Distance Strategy (PDS), in which partial distances are computed among all available resources and then re-scaled by the reciprocal of the proportion of observed values. The third technique, called Optimal Completion Strategy (OCS), computes missing values iteratively as auxiliary variables in the optimization of a suitable objective function.

In the evaluation of the VKFCM-K-LP method under the WDS, PDS and OCS approaches, we considered artificially generated datasets with 5%, 10%, 15% and 20% of missing values. The results of the analyzes were quantified according to the following quality measures: the Corrected Rand index (CR), F*-measure* (FM), the Overall Error Rate of Classification (OERC) and the measure of consistency of variables for the OCS [47–50]. In addition, the results of the clustering under OCS were compared with the results of the clustering using the imputation methods via the mean and the median values.

The rest of the paper is structured as follows. In Section 2 the basic theory about kernels is briefly presented. Section 3 describes the conventional kernel fuzzy *C*-means (KFCM) algorithm under the kernelization of the metric approach. Section 4 presents the kernel-based fuzzy clustering with variable weighting via local adaptive distances under the kernelization of the metric approach (VKFCM-K-LP). Section 5 introduces the main approach to analyze missing data. New VKFCM-K-LP algorithms under the WDS, PDS and OCS schemes are proposed in Section 6. Section 7 proposes the experimental design. Section 8 contains the results of several numerical evaluations. Finally, Section 9 offers some concluding remarks.

## 2 Theoretical background

This section describes the basic theory about kernels. The main idea behind kernel-based methods is the use of an arbitrary nonlinear mapping *ϕ* from the original space of the input data to a space of higher dimension (possibly infinite), called feature space F.

Let *X* = {**x**_1_, **x**_2_, …, **x**_*n*_} be a non-empty set with $x_{i}\in R^{p},\forall_{i}$. A function K:X×X→R is a Mercer Kernel, if and only if, K is symmetric, i.e. $K\left(x_{k},x_{i}\right)=K\left(x_{i},x_{k}\right)$ and the following inequality is valid [29]:

$$\begin{array}{c} \sum_{i=1}^{n}\sum_{k=1}^{n}c_{i}c_{k}K\left(x_{i},x_{k}\right)\geq 0,\;\;\forall_{n}\geq 2; \end{array}$$ (1)

where, $c_{r}\in R,\;\forall r=1,\ldots ,n$. Each Mercer Kernel can be expressed as:

$$\begin{array}{c} K\left(x_{i},x_{k}\right)=\phi {\left(x_{i}\right)}^{⊤}\phi \left(x_{k}\right), \end{array}$$ (2)

in which, ϕ:X→F performs a nonlinear mapping from the original space of *X* to the space of high-dimensional features F.

One of the most relevant aspects in the application of Kernel-based methods is the possibility to calculate Euclidean distances in F without having to explicitly specify the non-linear mapping *ϕ* [51, 52].

This can be done using the so called distance Kernel trick [52, 53]:

$$\begin{array}{ll} ∥\phi (x_{i})-\phi (x_{k}){∥}^{2} & ={\left(\phi (x_{i})-\phi (x_{k})\right)}^{⊤}(\phi (x_{i})-\phi (x_{k}))\\ & =\phi {\left(x_{i}\right)}^{⊤}\phi (x_{i})-2\phi {\left(x_{i}\right)}^{⊤}\phi (x_{k})+\phi {\left(x_{k}\right)}^{⊤}\phi (x_{k})\\ & =K(x_{i},x_{i})-2K(x_{i},x_{k})+K(x_{k},x_{k}), \end{array}$$ (3)

where, the calculation of the distances in the feature space is a function of the input vectors. Kernel functions [54] typically used are:

- Linear: $K\left(x_{i},x_{k}\right)=x_{i}^{⊤}x_{k}$,
- Polynomial of degree *d*: $K\left(x_{i},x_{k}\right)={\left(\gamma x_{i}^{⊤}x_{k}+\theta \right)}^{d}$, **γ** > 0, *θ* > 0, d∈N,
- Gaussian: $K\left(x_{i},x_{k}\right)=e^{-\frac{\|x_{i}-x_{k}{\|}^{2}}{2\sigma^{2}}}$, *σ* > 0,
- Laplacian: $K\left(x_{i},x_{k}\right)=e^{-\gamma \|x_{i}-x_{k}\|}$, *γ* > 0,

where, *γ*, *θ*, *σ* and *d* are Kernel parameters. In the literature, Kernel-based clustering methods can be divided into two main categories, kernelization of the metric [16, 55] and clustering in feature space [56]. However, in this work, we consider only the kernelization of the metric approach. Under this approach, clustering methods seek for prototypes in the original space of the input data and the distances between a data point **x**_*i*_ and the prototype of the *k*-th group **v**_*k*_ are obtained by means of kernel functions:

$$∥\phi (x_{i})-\phi (v_{k}){∥}^{2}=K(x_{i},x_{i})-2K(x_{i},v_{k})+K(v_{k},v_{k}).$$ (4)

## 3 Kernel fuzzy *C*-means (KFCM)

Let Ω = {1, …, *n*} be a set of *n* observations indexed by *i* and described by *p* variables. Let *P* = {*P*_1_, *P*_2_, …, *P*_*k*_} be a partition of Ω in *K* groups. The purpose of the Kernel fuzzy C-Means clustering method under kernelization of the metric is to minimize the following objective function

$$J=\sum_{k=1}^{K}\sum_{i=1}^{n}{\left(u_{ki}\right)}^{m}∥\varphi (x_{i})-\varphi (v_{k}){∥}^{2},\;\text{subject}\;\text{to,}\;\{\begin{array}{ll} u_{ki}\in [0,1], & \forall k,i,\\ \sum_{k=1}^{K}u_{ki}=1, & \forall i, \end{array}$$ (5)

where $v_{k}\in R^{p}$ is the prototype of the *k*-th cluster, *k* = 1, …, *K*, *u*_*ki*_ is the fuzzy membership degree of the observation *i* to the *k*-th cluster, *k* = 1, …, *K*, *i* = 1, …, *n* and $m\in R^{+}$ is a parameter that controls the fuzziness of the membership for each observation *i*. Here, $U=\left[u_{ki}\right]\in R^{K\times n}$ is the fuzzy partition matrix. Deriving prototypes for the clusters depends on the choice of the kernel function. When considering the Gaussian Kernel, the most popular in literature, we have that $K(x_{i},x_{i})=1$, for all *i* = 1, …, *n*. Thus, the objective function described in Eq (5) can be expressed as in Graves et al. [57] by Eq (6):

$$\begin{array}{c} J=2\sum_{k=1}^{K}\sum_{i=1}^{n}{\left(u_{ki}\right)}^{m}\left(1-K\left(x_{i},v_{k}\right)\right), \end{array}$$ (6)

therefore the equation of the cluster prototypes is defined for *k* = 1, …, *K* as

$$\begin{array}{c} v_{k}^{\left(t+1\right)}=\frac{\sum_{i=1}^{n}{\left(u_{ki}^{\left(t+1\right)}\right)}^{m}K\left(x_{i},v_{k}^{\left(t\right)}\right)x_{i}}{\sum_{i=1}^{n}{\left(u_{ki}^{\left(t+1\right)}\right)}^{m}K\left(x_{i},v_{k}^{\left(t\right)}\right)}. \end{array}$$ (7)

When updating the fuzzy partition matrix **U**, the prototypes **v**_*k*_ are kept fixed and we need to find the fuzzy membership degrees *u*_*ki*_ (*k* = 1, …, *K*, *i* = 1, …, *n*). Using the Lagrange multipliers for the optimization process of the objective function *J*, subject to the restrictions in Eq (5), we have the following solution [57]:

$$\begin{array}{c} u_{ki}^{\left(t+1\right)}\;\;=\;\;{\left[\sum_{h=1}^{K}{\left(\frac{1-K(x_{i},v_{k}^{\left(t+1\right)})}{1-K(x_{i},v_{h}^{\left(t+1\right)})}\right)}^{\frac{1}{m-1}}\right]}^{-1}\;\;\;\;. \end{array}$$ (8)

## 4 Kernel-based fuzzy clustering with automatic variable weighting via local adaptive distance

Kernel-based clustering methods commonly found in the literature, such as the kernel *Fuzzy C-Means* [58], do not take into account the weights or the relevance of each variable in the clustering process. However, for the majority of the datasets, and especially if we are dealing with high-dimensional data, some variables may be irrelevant, and, among the relevant variables, some may present greater or lesser importance than others. Moreover, different groups can have different sets of relevant variables. Motivated by this problem, Ferreira et al. [13] proposed a family of kernel-based fuzzy clustering methods with automatic weighting of variables, which are clustering algorithms in which dissimilarity measures are obtained as sums of Euclidean distances between patterns and cluster prototypes computed separately for each variable. The main idea supporting these methods is that the sum of kernel functions applied on each variable is also a kernel function. This reasoning enables the introduction of weights representing the relevance of each variable.

The clustering method VKFCM-K-LP takes into account the weights or the relevance of each variable for the construction of the clusters [13]. This clustering method is based on a kernelized local adaptive distance with the constraint that the product of the weights of the variables on each cluster must be equal to 1. The algorithm considers a separate weight vector for each cluster in order to parameterize its local distances. Then, the closer the observations are to the prototype of a given cluster with respect to a given variable, the greater its importance to this cluster. The restrictions on the weight vector in the VKFCM-K-LP method are based on hard clustering via adaptive distances and on *fuzzy* quadratic distances [59, 60].

**Result 1** (**Scholkopf and Smola** [53]) *If* $K_{1}:X_{1}\times X_{1}\to R$ *and* $K_{2}:X_{2}\times X_{2}\to R$ *are kernel functions, then the sum*, $K\left(x_{1},x_{1}^{\prime }\right)+K\left(x_{2},x_{2}^{\prime }\right)$ *is a kernel function defined in* (*X*_1_ × *X*_2_) × (*X*_1_ × *X*_2_), *where* $x_{1},x_{1}^{\prime }\in X_{1}$, $x_{2},x_{2}^{\prime }\in X_{2}$ *and* $X_{1},X_{2}\subset R^{p}$.

Under this result, if an instance is represented by a vector with *p* variables, we can partition it into up to *p* parts, and consider up to *p* different kernel functions, one for each part. Formally, we have that $K(x_{i},x_{k})=\sum_{j=1}^{p}K_{j}\left(x_{ij},x_{kj}\right)$, where $K_{j}:X_{j}\times X_{j}\to R$ are Kernel functions and *X*_*j*_ is the the space of the *j*-th variable with *j* = 1, …, *p*. Therefore, a distance based on kernelizing the metric between an instance **x**_*i*_ and the *k*-th prototype **v**_*k*_ with respect to the *j*-th variable [51, 52] is defined by

$${\left\|\phi_{j}(x_{ij})-\phi_{j}(v_{kj})\right\|}^{2}=K_{j}(x_{ij},x_{ij})-2K_{j}(x_{ij},v_{kj})+K_{j}(v_{kj},v_{kj}),$$ (9)

in which *ϕ*_*j*_ *j* = 1, …, *p* is a non-linear mapping of **x**_*i*_ ∈ *X*, $X\subset R^{p}$ into the feature space $F_{j}$ concerning the *j*-th variable. In Eq (9) it is possible to introduce weights representing the relevance of each variable. Let *φ*^2^(**x**_*i*_, **v**_*k*_) be a distance measure based on kernelization of the metric between an observation **x**_*i*_ and the prototype **v**_*k*_ of the *k*-th cluster. Thus, the local adaptive distance *φ*^2^(**x**_*i*_, **v**_*k*_) with the restriction that the product of the weights of the variables in each cluster [61] is equal to 1, is given by

$$\begin{array}{c} \varphi_{\lambda_{k}}^{2}\left(x_{i},v_{k}\right)=\sum_{j=1}^{p}\lambda_{kj}\|\phi_{j}\left(x_{ij}\right)-\phi_{j}\left(v_{kj}\right){\|}^{2}\text{,}\;\text{subject}\;\text{to}\;\{\begin{array}{cc} \lambda_{kj}>0, & \forall i,j,\\ \prod_{j=1}^{p}\lambda_{kj}=1, & \forall k, \end{array} \end{array}$$ (10)

where **λ**_*k*_ = (λ_*k*1_, …, λ_*kp*_) is the vector of weights for the *k*-th cluster. Given Eqs (9) and (10) we can define an objective function *J* that measures the fit between the clusters and their prototypes, given by

$$\begin{array}{c} J=\sum_{k=1}^{K}\sum_{i=1}^{n}{\left(u_{ki}\right)}^{m}\varphi^{2}\left(x_{i},v_{k}\right)=\sum_{k=1}^{K}\sum_{i=1}^{n}{\left(u_{ki}\right)}^{m}\sum_{j=1}^{p}\lambda_{kj}\|\phi_{j}\left(x_{ij}\right)-\phi_{j}\left(v_{kj}\right){\|}^{2}, \end{array}$$ (11)

subject to the constraints given in Eq (5), where *u*_*ki*_ is the fuzzy membership degree for observation *i* in the *k*-th cluster *k* = 1, …, *K*, *i* = 1, …, *n* and $v_{k}\in R^{p}$ is the prototype of the *k*-th cluster.

When considering the Gaussian Kernel the objective function described in the Eq (11) is rewritten as

$$\begin{array}{c} J=2\sum_{k=1}^{K}\sum_{i=1}^{n}{\left(u_{ki}\right)}^{m}\sum_{j=1}^{p}\lambda_{kj}\left(1-K\left(x_{ij},v_{kj}\right)\right). \end{array}$$ (12)

While deriving cluster prototypes, the fuzzy membership degrees and the weights of the variables are kept fixed. Therefore, the prototype of the *k*-th cluster **v**_*k*_ = (*v*_*k*1_, …, *v*_*kp*_) (*k* = 1, …, *K*) that minimize criterion *J* in Eq (12) has its components *v*_*kj*_ (*j* = 1, …, *p*) defined by

$$\begin{array}{c} v_{kj}^{\left(t+1\right)}=\frac{\sum_{i=1}^{n}{\left(u_{ki}^{\left(t+1\right)}\right)}^{m}K_{j}\left(x_{ij},v_{kj}^{\left(t\right)}\right)x_{ij}}{\sum_{i=1}^{n}{\left(u_{ki}^{\left(t+1\right)}\right)}^{m}K_{j}\left(x_{ij},v_{kj}^{\left(t\right)}\right)}. \end{array}$$ (13)

in which, *t* = 1, …, *T* where *T* is the maximum number of iterations. The next step is to determine the weights of the variables. To do so, the fuzzy membership degrees *u*_*ki*_ and the cluster prototypes **v**_*k*_ are kept fixed. The weight vector **λ**_*k*_ = (λ_*k*1_, …, λ_*kp*_) that minimizes criterion *J*, under restrictions λ_*kj*_ > 0 ∀_*kj*_ and $\prod_{j=1}^{p}\lambda_{kj}=1$, ∀_*k*_, has its components λ_*kj*_ (*j* = 1, …, *p*, *k* = 1, …, *K*) given by

$$\begin{array}{c} \lambda_{kj}^{\left(t+1\right)}\;\;=\;\frac{\prod_{l=1}^{p}{\left\{\sum_{i=1}^{n}{\left(u_{ki}^{\left(t+1\right)}\right)}^{m}\|\phi \left(x_{il}\right)-\phi \left(v_{kl}^{\left(t+1\right)}\right){\|}^{2}\right\}}^{\frac{1}{p}}}{\sum_{i=1}^{n}{\left(u_{ki}^{\left(t+1\right)}\right)}^{m}\|\phi \left(x_{ij}\right)-\phi \left(v_{kj}^{\left(t+1\right)}\right){\|}^{2}}. \end{array}$$ (14)

While updating the fuzzy membership degrees, the prototypes of the clusters **v**_*k*_ and the weights of the variables are kept fixed. Therefore, the fuzzy membership degrees that minimize criterion *J*, given in Eq (5), are updated according to the following expression

$$\begin{array}{c} u_{ki}^{\left(t+1\right)}={\left[\sum_{h=1}^{K}{\left(\frac{\varphi^{2}\left(x_{i},v_{k}^{\left(t+1\right)}\right)}{\varphi^{2}\left(x_{i},v_{h}^{\left(t+1\right)}\right)}\right)}^{\frac{1}{m-1}}\right]}^{-1}, \end{array}$$ (15)

where *φ*^2^(**x**_*i*_, **v**_*k*_) is defined in Eq (10). Algorithm 1 shows the steps of the VKFCM-K-LP method. The convergence properties of the method were demonstrated in the work of [13].

**Algorithm 1**: VKFCM-K-LP clustering method

1: Initialization

 Fix *K* (number of clusters), 2 ≤ *K* < *n*; fix *m*, 1 < *m* < ∞; fix *T* (number of iterations);

 and fix *ϵ*, 0 < *ϵ* < 1. Randomly initialize the fuzzy membership degrees *u*_*ki*_ with the restrictions given in Eq (5);

 Uniformly initialize all weights as 1/*p*.

 Do *t* = 1.

2: Update prototype vector **v**_*k*_ according to Eq (13).

3: Update weight vector **λ**_*k*_ according to Eq (14).

4: Update fuzzy membership degree *u*_*ki*_

 given in Eq (15).

5: IF |*J*^*t*+1^ − *J*^*t*^| ≤ *ϵ* or *t* > *T*

 STOP

 ELSE do *t* = *t* + 1 and go to step 2.

## 5 Incomplete data analysis

Data quality is one of the most important factors that can affect the results of statistical analysis. Problems during data collection or pre-processing can generate uncertain values, incorrect or even absent values. Data analysis with missing data is a problem often discussed in many areas of science, because these analyses were originally designed for datasets without missing values. Although the causes of missing data are diverse in the literature, there are few missing data patterns resulting from the missing values in the datasets. The missing data pattern describes which values are observed and which values are absent from the dataset [35].

**Fig 1:**
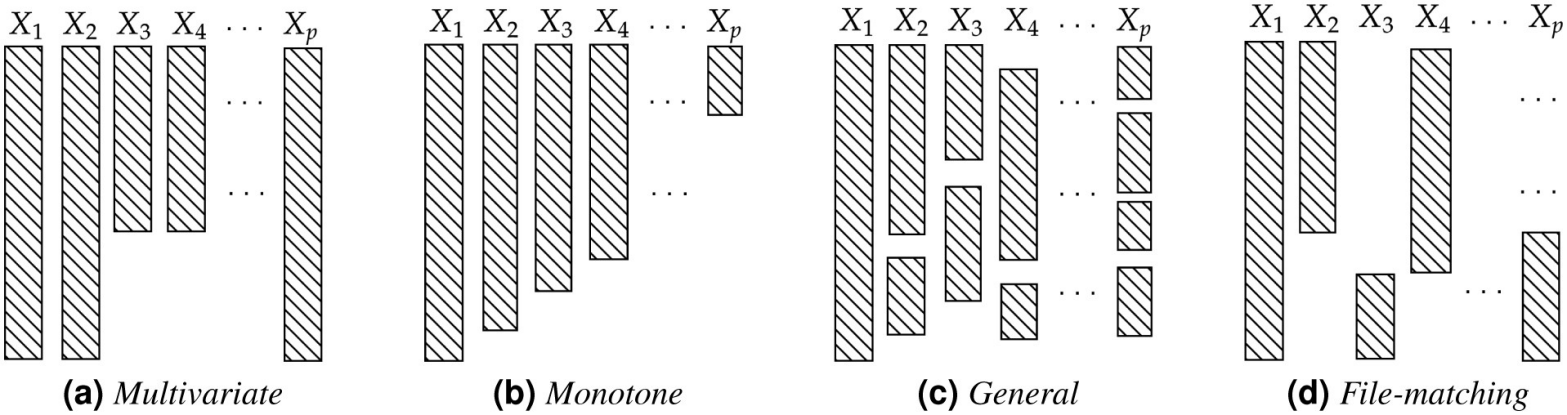
Types of missing data patterns. (**a**) *Multivariate*. (**b**) *Monotone*. (**C**) *General*. (**d**) *File-matching*.

Generally, the most common missing data patterns are the multivariate, monotone, general and file-matching patterns [35]. In the multivariate pattern (Fig 1a), missing values occur in a group of attributes that are completely observed or missing. The monotone pattern (Fig 1b) usually occurs as a result of longitudinal studies and has a ladder-like arrangement of values when organized in a data matrix. The file-matching pattern (Fig 1d) occurs when the data are obtained from several different sources and, consequently, the combined dataset will have fully observed attributes and features that are not jointly observed.

In the general pattern (Fig 1c) the missing values are characterized by an arbitrary form in the dataset and can be observed in practice for example, in the omission of responses in a questionnaire or loss of data in pre-processing.

Although missing data patterns describe what values are missing from the dataset, missing data generation mechanisms provide information about the occurrence of these values. Missing data generation mechanisms refer to the relationship between the missing value and the attribute values of the variables in the dataset. Therefore, whereas a missing data pattern indicates what values in the dataset can be used for statistical analysis, mechanisms provide an indication of how the available values should be treated during data analysis to obtain the best results.

The first works that deal with missing data generation mechanisms were proposed by Rubin [34] and are still used today. These mechanisms are known as: Missing Completely at Random (MCAR), Missing at Random (MAR) and Not Missing at Random (NMAR) and describe the relationship between the analyzed variables and the percentage of missing values in the data matrix [62, 63]. In this work, we focus on strategies for dealing with missing data of the MCAR type [35]. Let *X* = {**x**_1_, …, **x**_*n*_} be a data matrix and define the *p*-dimensional vector **x**_*i*_ = {*x*_*i*1_, *x*_*i*2_, …, *x*_*ip*_}, for 1 ≤ *i* ≤ *n* and 1 ≤ *j* ≤ *p*, where *x*_*ij*_ is the *j*-th variable of the *i*-th observation. We can rewrite *X* as *X* = *X*_*obs*_ ∪ *X*_*M*_, where *X*_*obs*_ = {*x*_*ij*_}, if this value is observed in *X*, and *X*_*M*_ = {*x*_*ij*_ = *NA*} if this value is missing in *X*. In this context, we define a missing indicator matrix **M** = [*m*_*ij*_] that shows if the observation value *x*_*ij*_ is missing (*m*_*ij*_ = 1) or if *x*_*ij*_ is observed (*m*_*ij*_ = 0). The missing data generation mechanism is defined as the conditional probability of **M** given *X*, *P*(**M**|*X*, *θ*), where *θ* denotes the unknown parameters of a given probability distribution. Missing values are defined as MCAR if a missing value does not depend on the dataset. Formally, this mechanism is defined as:

$$\begin{array}{c} P\left(M|X,\theta \right)=P\left(M|\theta \right),\;\text{for}\;\text{all}\;x_{ij}\in X,\theta . \end{array}$$ (16)

From a practical perspective, missing data mechanisms operate as assumptions that dictate which techniques should be used to deal with these values [62].

### 5.1 Handling missing values

Traditionally, researchers use a wide variety of techniques to handle missing values. However, the best method would be to avoid having these values in the dataset, through better experiment mapping or repeated data collection. Nonetheless, investigating why these values are absent and taking corrective measures can become impracticable or impossible. Therefore, it is usually more feasible to adopt techniques that deal with missing values in the data matrix. There are three common approaches in the literature to manipulate missing values [35]:

- **Elimination**: This technique is best used when the percentage of missing values in the dataset is relatively small. The approach is to ignore missing data items or the attributes that contain those values. Therefore, data analysis is performed on the set of available data, called Complete-Case Analysis (CCA). The main advantage of exclusion is that it produces a complete dataset, which in turn allows the use of standard data analysis techniques [62]. The disadvantage of this technique is that the sample size can be drastically reduced, especially for datasets that include a large proportion of missing data.
- **Imputation**: This approach, which is called Imputation of Missing Values (IMV), consists of replacing the missing values with estimated values that are generally derived from the available data. IMV techniques range from simple methods, such as replacing missing values with the Mean or the Median value, to more sophisticated ones that use Regression, Maximum Likelihood and other statistical methods [63]. The disadvantage of this approach is that the quality of the results of the data analysis can be affected by the imputation, since imputed values are treated as observed values. As an advantage, standard analysis techniques can be used since the missing values have been filled.
- **Adaptation of data analysis methods to incomplete data**: An effective approach is to adapt data analysis methods so that they can handle datasets that have missing values. These methods include estimating missing values during data analysis and distinguishing between observed and imputed values. The main advantage of the adaptation approach is that all observed data can be used for data analysis, avoiding the disadvantages of imputing the missing values.

## 6 Adapting the VKFCM-K-LP algorithm to handle missing data

The VKFCM-K-LP clustering method [13] cannot be applied directly to datasets with missing values. As with most clustering methods, VKFCM-K-LP requires all values in the data matrix to be present, in order to calculate prototypes and distance measurements. Several methods have been proposed in the literature to deal with incomplete data, such as Hathaway et al. [43], who proposed three strategies to group incomplete data using the Fuzzy *C-Means* algorithm (FCM). In this Section, we use these three approaches to adapt the VKFCM-K-LP clustering algorithm to deal with incomplete data.

### 6.1 Whole Data Strategy (WDS)

This strategy consists of omitting the incomplete data items and applying the VKFCM-K-LP algorithm to the resulting complete data matrix [43]. This method is an example of CCA, since the missing values are not included in the calculation of the cluster prototypes, and can be applied when the percentage of missing data is relatively small. It is generally suggested that WDS can be considered when the percentage of missing values is less than 25% of all values in the dataset [43]. However, incomplete observations are not completely excluded from the analysis. At the end of the clustering process using the complete dataset, incomplete data are partitioned using the nearest-prototype scheme based on Partial Distances (PD) computed from each incomplete instance to each cluster prototype. The PD function calculates the sum of the squared (kernelized) Euclidean distances between all available observations (i.e. non-missing) and then weights them by the proportion of values used in their calculation. Algorithm 2 describes the steps for WDS.

**Algorithm 2**: VKFCM-K-LP clustering method with the WDS strategy.

1: Initialization

 Fix *K* (number of clusters), 2 ≤ *K* < *n*; fix *m*, 1 < *m* < ∞;

 fix *T* (number of iterations); and fix *ϵ*, 0 < *ϵ* < 1.

 Randomly initialize the fuzzy membership degrees *u*_*ki*_;

 Uniformly initialize all weights as 1/*p*.

 Do *t* = 1.

2: Update prototype vector **v**_*k*_ according to Eq (13).

3: Update weight vector **λ**_*k*_ according to Eq (14).

4: Update fuzzy membership degree *u*_*ki*_ using Eq (15).

5: IF |*J*^*t*+1^ − *J*^*t*^| ≤ *ϵ* OR *t* > *T*

 Partition *X*_*M*_ according to Eq 17

 STOP

 ELSE do *t* = *t* + 1 and go to step 2.

### 6.2 Partial Distance Strategy (PDS)

Dixon [64] recommends the partial distance strategy in cases when *X*_*M*_ is sufficiently large and WDS cannot is not recommended. PDS consists of estimating the distance between two observations using the Partial Distance function. In VKFCM-K-LP, which uses a local adaptive kernel distance, its partial version is given by

$$\begin{array}{c} \varphi_{dp}^{2}\left(x_{i},v_{k}\right)=\frac{p}{I_{i}}\sum_{j=1}^{p}\lambda_{kj}{\left\|\phi \left(x_{ij}\right)-\phi \left(v_{kj}\right)\right\|}^{2}I_{ij}, \end{array}$$ (17)

where $I_{i}=\sum_{j=1}^{p}I_{ij}$ for 1 ≤ *i* ≤ *n* and 1 ≤ *j* ≤ *p*. The indicator function *I*_*ij*_ is defined by

$$\begin{array}{c} I_{ij}=\{\begin{array}{cc} 1, & \text{if}\;x_{ij}\in X_{obs},\\ 0, & \text{if}\;x_{ij}\in X_{M}. \end{array} \end{array}$$ (18)

where *X*_*obs*_ and *X*_*M*_ are defined in Section 5. Therefore the objective function for this strategy is given by

$$\begin{array}{c} J_{pd}\left(V,U,\Lambda \right)=\sum_{k=1}^{K}\sum_{i=1}^{n}{\left(u_{ki}\right)}^{m}\varphi_{pd}^{2}\left(x_{i},v_{k}\right), \end{array}$$ (19)

where $V=\left\{v_{1},\ldots ,v_{K}\right\}\in R^{K\times p}$, $\Lambda =\left\{\lambda_{1},\ldots ,\lambda_{K}\right\}\in R_{+}^{K\times p}$ and $\varphi_{dp}^{2}\left(x_{i},v_{k}\right)$, which is defined in Eq (17), is called Local Adaptive Partial Kernel with the constraint given in Eq (10).

In the first iteration of the VKFCM-K-LP algorithm, prototypes and weights are updated using only the values in *X*_*obs*_. Prototypes are given by

$$\begin{array}{c} v_{kj}^{\left(t+1\right)}=\frac{\sum_{k=1}^{n}{\left(u_{ik}^{\left(t+1\right)}\right)}^{m}K\left(x_{ij},v_{kj}^{\left(t\right)}\right)x_{ij}I_{ij}}{\sum_{i=1}^{n}{\left(u_{ki}^{\left(t+1\right)}\right)}^{m}K\left(x_{ij},v_{kj}^{\left(t\right)}\right)I_{ij}}, \end{array}$$ (20)

where K(.) is the Gaussian Kernel. The weights of the variables are obtained by minimizing the objective function given in Eq (19), which gives Eq (21).

$$\begin{array}{c} \lambda_{kj}^{\left(t+1\right)}\;\;=\frac{\prod_{l=1}^{p}\{\sum_{i=1}^{n}{\left(u_{ki}^{\left(t+1\right)}\right)}^{m}{\left\|\phi \left(x_{il}\right)-\phi \left(v_{kl}^{\left(t+1\right)}\right)\right\|}^{2}I_{il}{\}}^{\frac{1}{p}}}{\sum_{i=1}^{n}{\left(u_{ki}^{\left(t+1\right)}\right)}^{m}{\left\|\phi \left(x_{ij}\right)-\phi \left(v_{kj}^{\left(t+1\right)}\right)\right\|}^{2}I_{ij}}, \end{array}$$ (21)

for 1 ≤ *k* ≤ *K* and 1 ≤ *l* ≤ *p*. The scale factor *p*/*I*_*i*_ in Eq (17) has no effect on the calculation of prototypes [43] in Eq (20) and consequently it does not affect the weight calculation in Eq (21). This scale factor also has no effect on *u*_*ki*_, which is calculated using Eq (22), because it appears both at the top and at the bottom of the equation and can be omitted from the partial distance given in Eq (17).

$$\begin{array}{c} u_{ki}^{\left(t+1\right)}={\left[\sum_{h=1}^{K}{\left(\frac{\varphi_{dp}^{2}\left(x_{i},v_{k}^{\left(t+1\right)}\right)}{\varphi_{dp}^{2}\left(x_{i},v_{h}^{\left(t+1\right)}\right)}\right)}^{\frac{1}{m-1}}\right]}^{-1} \end{array}$$ (22)

The steps of the PDS version VKFCM-K-LP are listed in Algorithm 3.

**Algorithm 3**: VKFCM-K-LP clustering method with the PDS strategy.

1: Initialization

 Fix *K* (number of clusters), 2 ≤ *K* < *n*;

 Fix *m*, 1 < *m* < ∞; fix *T* (number of iterations); and fix *ϵ*, 0 < *ϵ* < 1.

 Randomly initialize the fuzzy membership degrees *u*_*ki*_;

 Uniformly initialize all weights with 1/*p*.

 Do *t* = 1.

2: Update prototype vector **v**_*k*_, according to Eq (20).

3: Update weight vector **λ**_*k*_ according to Eq (21).

4: Update fuzzy membership degree *u*_*ki*_ using Eq (22).

5: IF |*J*^*t*+1^ − *J*^*t*^| ≤ *ϵ* OR *t* > *T*

 STOP

 ELSE do *t* = *t* + 1 and got to the step 2.

### 6.3 Optimal Completion Strategy (OCS)

The main idea of this strategy is to iteratively calculate the missing values in *X*_*M*_ as auxiliary variables in the optimization of the objective function *J*_*M*_ [43] defined in Eq (23).

$$\begin{array}{c} J_{M}\left(V,U,\Lambda ,X_{M}\right)=\sum_{k=1}^{K}\sum_{i=1}^{n}{\left(u_{ki}\right)}^{m}\varphi_{\lambda_{k}}^{2}\left(x_{i},v_{k}\right), \end{array}$$ (23)

in which

$$\begin{array}{ll} \varphi^{2}(x_{i},v_{k}) & =\sum_{j=1}^{p}\lambda_{kj}{∥\varphi (x_{ij})-\varphi (v_{kj})∥}^{2}\\ & =2\sum_{j=1}^{p}\lambda_{kj}(1-K(x_{ij},v_{kj})). \end{array}$$ (24)

Prototype *v*_*kj*_ and weight λ_*kj*_ are defined according to Eqs (13) and (14). Thus, the missing values are updated by minimizing Eq (25).

$$\begin{array}{c} X_{M}^{\left(t+1\right)}=\underset{X_{M}}{\text{arg}\;\text{min}}\left\{J_{M}\left(U^{\left(t+1\right)},V^{\left(t+1\right)},\Lambda^{\left(t+1\right)},X_{M}^{\left(t\right)}\right)\right\}. \end{array}$$ (25)

Thus, the missing value *x*_*ij*_ ∈ *X*_*M*_ is given by Eq (26) as described in [43].

$$\begin{array}{c} x_{ij}^{\left(t+1\right)}=\frac{\sum_{k=1}^{K}{\left(u_{ki}^{\left(t+1\right)}\right)}^{m}v_{kj}^{\left(t+1\right)}}{\sum_{k=1}^{K}{\left(u_{ki}^{\left(t+1\right)}\right)}^{m}}, \end{array}$$ (26)

where membership degree *u*_*ki*_ is defined as in Eq (15) and 1 ≤ *i* ≤ *n* and 1 ≤ *j* ≤ *p*. In this strategy, missing values are imputed by the weighted averages of all prototypes at each iteration. Moreover the missing values *X*_*M*_ are initialized using random values. The expression in Eq (26) is obtained through the partial derivatives of the objective function given in Eq (23), by fixing prototypes, weights and memberships. Algorithm 4 describes the steps of the VKFCM-K-LP method under the OCS approach. The advantage of this approach is that the missing values are allocated during the clustering process.

**Algorithm 4**: VKFCM-K-LP clustering method with the OCS strategy.

1: Initialization

 Fix *K* (number of clusters), 2 ≤ *K* < *n*; fix *m*, 1 < *m* < ∞;

 fix *T* (number of iterations); fix *ϵ*, 0 < *ϵ* < 1.

 Randomly initialize *X*_*M*_;

 Randomly initialize the fuzzy membership degrees *u*_*ki*_ with the restrictions given in (5);

 Uniformly initialize all weights as 1/*p*;

 Do *t* = 1.

2: Update prototype vector **v**_*k*_ according to Eq (13).

3: Update weight vector **λ**_*k*_ according to Eq (14).

4: Update fuzzy membership degree *u*_*ki*_ according to Eq (15).

5: Update *x*_*ij*_ ∈ *X*_*M*_ according to Eq (26)

6: IF |*J*^*t*+1^ − *J*^*t*^| ≤ *ϵ* OR *t* > *T*

 STOP

 ELSE do *t* = *t* + 1 and go to step 2.

## 7 Experimental design

The performance of the VKFCM-K-LP method proposed by [13] has not been evaluated in the context of incomplete data. Thus, this work adapted VKFCM-K-LP using the three strategies defined by [43] to handle missing data. To evaluate the methods, we implemented a missing value generator, in order to create reproducible datasets with absent values on which the methods presented in this work can be evaluated. The implementation of the missing data generation mechanism and the graphical representations were performed with the aid packages offered by R [65]. The main R packages used were ggplot2, VIM and naniar. The clustering methods were implemented using C. Experiments ran on an Intel Core (TM) I3-3217U CPU, clocking at 1.80GHz, with 4GB of RAM, using the Linux operating system. The code and data for reproducing the results here reported are available in the following repository: https://github.com/AnnyKerol/clustering_for_missing_data.

Three external indices were used to compare clustering results: Corrected Rand index (CR) [47], F-measure [48] and Overall Error Rate of Classification (OERC) [49]. The CR index takes its values from the interval [−1, 1], in which 1 indicates perfect agreement between partitions, whereas values near 0 (or negatives) correspond to cluster agreement found by chance [47]. F-measure takes its values from the [0, 1] interval, in which 1 indicates perfect agreement between partitions. OERC aims to measure the ability of a clustering algorithm to find original classes present in a dataset and takes its values from the [0, 1] interval, in which lower OERC values indicate better clustering results.

At the end of the clustering process of the VKFCM-K-LP method under the OCS approach, we obtained a complete dataset, which resulted in the best values of CR, OERC and F-measure. To verify if the values imputed by OCS resemble each variable’s distribution; we calculated a consistency measure [50] defined by

$$\begin{array}{c} d_{k}\left(j\right)=\frac{|\mu_{p0}\left(j\right)-\mu_{p1}\left(j\right)|}{\sqrt{\sigma_{p0}^{2}\left(j\right)+\sigma_{p1}^{2}\left(j\right)}}, \end{array}$$ (27)

where *k* denotes the *k*-th cluster, 1 ≤ *j* ≤ *p* and *p* represents the variables to be analyzed and *μ*_*p*0_ and $\sigma_{p0}^{2}$ are the mean and variance of the dataset with missing values, respectively. Additionally, *μ*_*p*1_ and $\sigma_{p1}^{2}$ refer to the mean and variance of the dataset with imputed values. The better the clustering under the OCS approach, the closer the values given by Eq (27) are to zero, which indicates that the imputed values were consistent in relation to the original scales of the variables in the dataset with missing values.

### 7.1 Missing data generation

The missing value generator used in this study removes values from the complete dataset with a given probability, according to the MCAR mechanism. In the generation of missing values of the MCAR type [35], we assume independence in the joint distribution of (**x**_*i*_, **M**), therefore, the probability that an *x*_*ij*_ value is observed is independent of the values in *X* or **M**. Consider a Bernoulli distribution with parameter *θ*, 0 ≤ *θ* ≤ 1, for the indicator variable **M**_*i*_, with probability P(**M**_i_ = 1|*x*_*i*_, *θ*), given that *x*_*i*_ is a missing value. If the missing values are independent from *X*, P(**M**_i_ = 1|*x*_*i*_, *θ*) = *θ*. Since the constant is independent of the values in *X*, this results in the generation of the MCAR type mechanism.

In computational terms, a complete dataset *X* is selected, and subsequently modified to obtain an incomplete dataset, by randomly selecting a specified percentage of its components {*x*_*ij*_} that are assigned as missing values. The {*x*_*ij*_} values are taken as missing when element *m*_*ij*_ from the sample generated for the indicator variable **M** is equal to one, i.e., *m*_*ij*_ = 1. Therefore the value of {*x*_*ij*_} is excluded from the complete dataset and designated as a missing value.

## 8 Results

This section presents an experimental evaluation of the kernel-based fuzzy clustering method with automatic weighting of the variables using local adaptive distances VKFCM-K-LP under the WDS, PDS and OCS approaches. In our experiments, datasets with 5%, 10%, 15% and 20% of missing values were artificially generated using the methodology described in Section 7.1, which means that random variable **M** was sampled from Bernoulli distributions with parameter *θ* taken from {0.05, 0.10, 0.15, 0.20}. The clustering algorithms were executed 100 times for each dataset, following a Monte Carlo simulation scheme with random initialization. On each Monte Carlo iteration, the adjustment between clusters and prototypes is observed until convergence, with a tolerance threshold of *ϵ* = 10^−10^ or until a maximum number of iterations is reached, i.e. until *t* > *T* with *T* = 300. At the end of the 100 Monte Carlo replications, we select the best solution according to objective function *J*.

In order to compare the models, we calculated CR, FM and OERC on their best solutions. The averages and standard deviations of these measures are also calculated across the 100 repetitions of each algorithm. The number of groups *K* was defined as equal to the known number of classes of each dataset. Parameter *m* was set as 2.0, following a previous study [13]. The terms $2\sigma_{j}^{2}$, {*j* = 1, …, *p*}, of the Gaussian Kernel functions, were estimated as the average between the 0.1 and 0.9 quantiles of ‖*x*_*ij*_ − *x*_*kj*_‖^2^ for *i* ≠ *k*; *i*, *k* = 1, …, *n* [13, 61].

Additionally, we calculated the consistencies of the variables in the complete datasets when evaluating the VKFCM-K-LP method under the OCS approach and we compared the clustering with the OCS method and the clustering using the imputation of missing values using Mean and Median values. To show the effectiveness of the VKFCM-K-LP clustering methods under the WDS, PDS and OCS approaches, we used two datasets: the *Iris Plant* dataset [66] and the *Thyroid Gland* dataset [67], both obtained from the Machine Learning Repository at the University of California, Irvine, United States (*UCI Machine Learning Repository*) [68]. The choice of these datasets is due to the fact that the groups have different structures, in particular the *Thyroid Gland* dataset presents greater group overlap than the *Iris Plant* dataset. The performances of the methods in these datasets are described in the following Sections.

### 8.1 *Iris Plant* dataset

The *Iris Plant* dataset [66] is well known and widely used in the area of pattern recognition. This set has three *a priori* classes (*K* = 3), each with 50 observations, for a total of 150 instances. The classes correspond to three species of Iris flowering plants: Iris setosa (Class 1), Iris virginica (Class 2) and Iris versicolor (Class 3). For each species, four variables were observed (*p* = 4), corresponding to flower measurements: Sepal Length (SL), Sepal Width (SW), Petal Length (PL) and Petal Width (PW).

**Fig 2:**
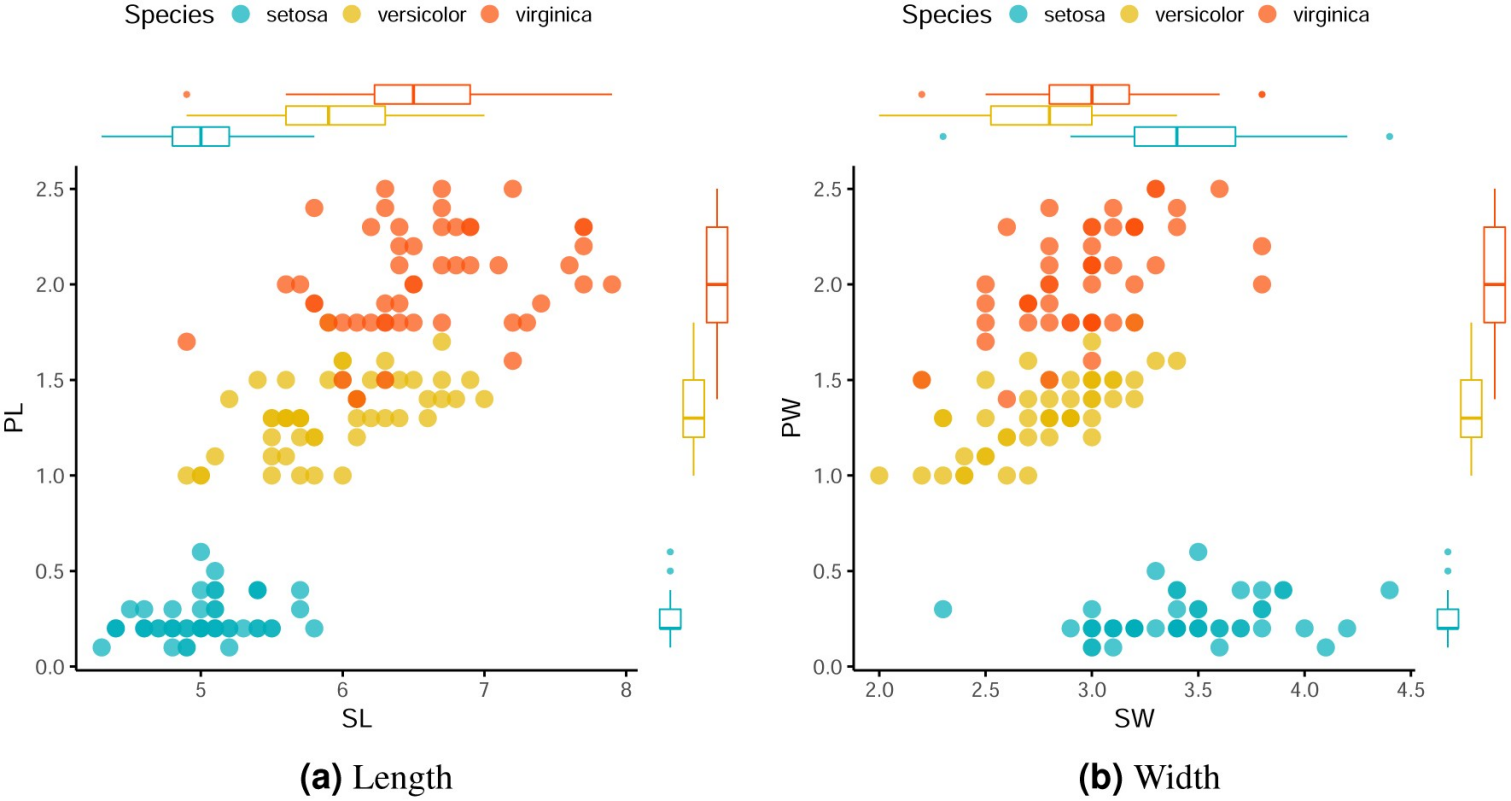
Scatter plots and boxplots for the *Iris Plant* dataset. (**a**) Length. (**b**) Width.

Fig 2a and 2b show the dispersion of the values of the variables for this dataset and the boxplots for each species. It is possible to observe an apparently linear relationship between variables PL and SL and between variables PW and SW for the versicolor and virginica classes. We also note that, considering the versicolor and virginica species, these variables are directly proportional, that is and increase in the value of SL implies an increase in the value of PL and the same is observed for SW and PW. In addition, the three species differ in relation to the variables, especially the setosa species, which is linearly separable from the other two.

The boxplots in Fig 2a show higher variability in the data of the virginica species for the SL and PL variables. Fig 2b, on the other hand, shows less variability when considering the SW variable.

**Fig 3:**
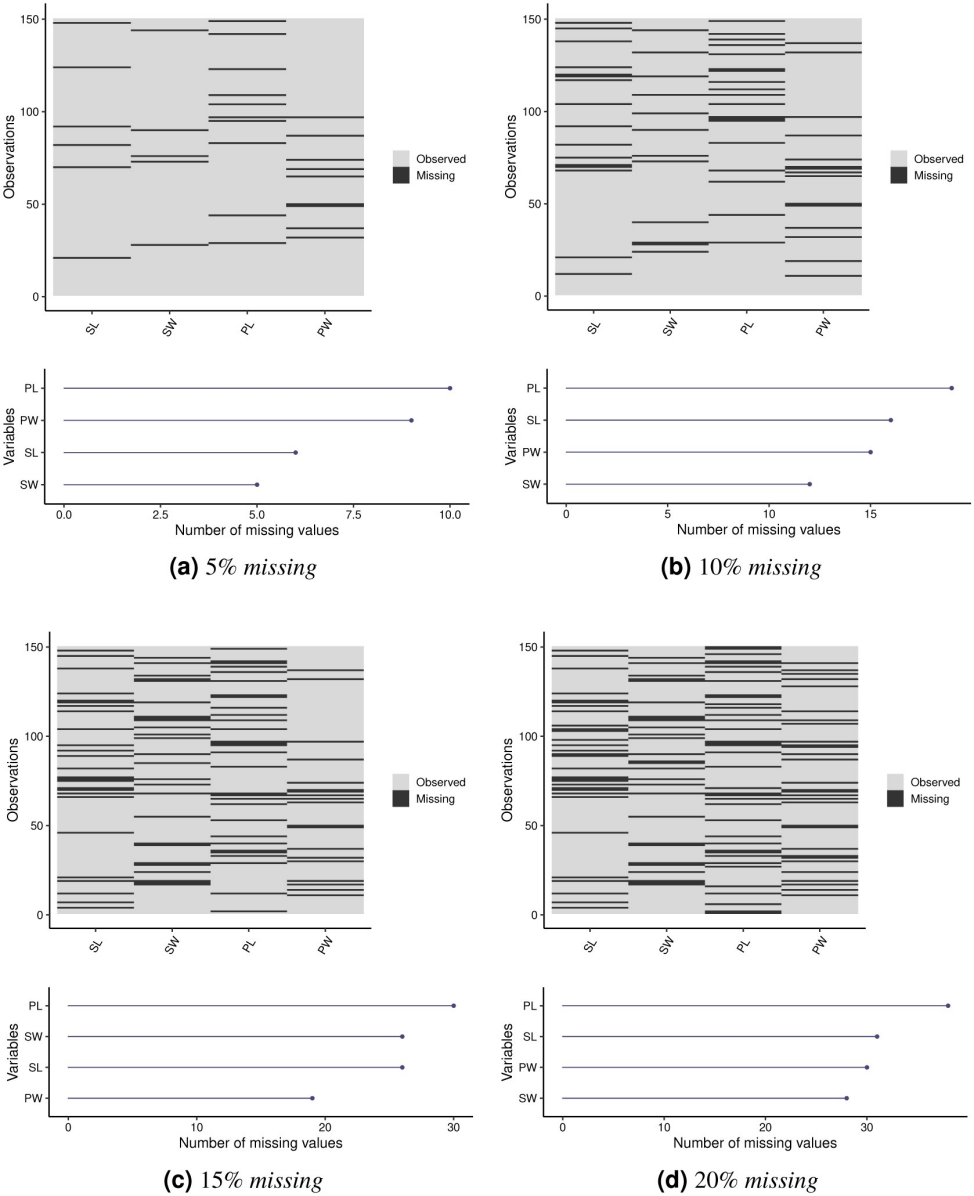
Visualizations of the patterns and frequencies of the missing values by variable for the *Iris Plant* dataset. (**a**) 5% *missing*. (**b**) 10% *missing*. (**c**) 15% *missing*. (**d**) 20% *missing*.

Fig 3a and 3b present the missing values patterns that were artificially generated for the *Iris Plant* dataset, distributed across its four variables. In each plot, the *x* axis represents the variables and the *y* axis represents the observations, with the black regions indicating missing values. The Figures also show the number of missing values by variable for each missing percentage, with variable PL having the highest number of missing values for all analyzed percentages. In datasets with 5%, 10% and 20% of missing values, the SW variable has the lowest missing amount. Observations belonging to Class 1 are in the 1|–50 range, while observations belonging to Class 2 are in the 51|–100 range, and, finally, the 101|–150 range represents observations belonging to Class 3.

Table 1 shows CR, FM and OERC corresponding to the best solutions obtained in the 100 Monte Carlo replications of the VKFCM-K-LP clustering algorithm with the WDS, PDS and OCS strategies. For all the missing value percentages studied, the CR and FM indices are close to 1, which indicates a good agreement between the *a priori* classes and the groups provided by the clustering methods. For 5% of missing values, the best performance was observed for the PDS method. However, when analyzing the data with 10%, 15% and 20% of missing values, the PDS method presented the worst performance. In general, increasing the percentage of missing values in the datasets affects the performance of the algorithms, as expected. This behavior is also verified for the PDS approach when increasing the percentage from 5% to 10% and for the WDS and OCS approaches when the percentage goes from 15% to 20%.

**Table 1:** Performance of the VKFCM-K-LP clustering algorithm with the WDS, PDS and OCS strategies for the dataset *Iris Plant*.

% NA	CR			FM			OERC		
	WDS	PDS	OCS	WDS	PDS	OCS	WDS	PDS	OCS
**5**	0.7429	0.8018	0.7861	0.8991	0.9261	0.9198	0.1000	0.0733	0.0800
**10**	0.8016	0.7561	0.8015	0.9266	0.9065	0.9266	0.0733	0.0933	0.0733
**15**	0.8176	0.7561	0.8175	0.9333	0.9065	0.9333	0.0666	0.0933	0.0666
**20**	0.8018	0.7561	0.7859	0.9261	0.9065	0.9199	0.0733	0.0933	0.0800

Aiming to investigate the predictive power of the VKFCM-K-LP algorithm under the three approaches for handling missing data, Table 2 shows the confusion matrices obtained for each method, and for each percentage of missing values considered.

**Table 2:** Confusion matrices obtained by the VKFCM-K-LP algorithm with the WDS, EDP and OCS strategies using 5%, 10%, 15% and 20% of missing values.

Methods	Clusters	5%			10%			15%			20%		
		1	2	3	1	2	3	1	2	3	1	2	3
WDS	1	50	0	0	50	0	0	50	0	0	50	0	0
	2	0	47	12	0	46	7	0	44	4	0	42	3
	3	0	3	38	0	4	43	0	6	46	0	8	47
PDS	1	50	0	0	50	0	0	50	0	0	50	0	0
	2	0	47	8	0	45	9	0	45	9	0	45	9
	3	0	3	42	0	5	41	0	5	41	0	5	41
OCS	1	50	0	0	50	0	0	50	0	0	50	0	0
	2	0	46	8	0	45	6	0	45	5	0	45	7
	3	0	4	42	0	5	44	0	5	45	0	5	43

In the columns we have the original classes, and in the lines we have the clusters provided by the clustering methods, which were identified as Cluster 1 (setosa), Cluster 2 (virginica) and Cluster 3 (versicolor).

The confusion matrices in Table 2 show that for all clustering methods and for all percentages of missing values considered, observations belonging to the setosa species in the dataset *Iris Plant* were properly grouped into Cluster 1. This is expected, as this species is separable from the other two species, as shown in Fig 2a and 2b. It can be also noted that Clusters 2 and 3 showed higher numbers of incorrectly clustered observations, which is expected because these groups are not linearly separable as observed for Cluster 1.

Tables 3–5 provide the weights of the variables in each cluster. In general, it is observed that in the three approaches and for all the percentages of missing values, variables PL and PW were the most relevant for the construction of the clusters. Variable PL obtained the greatest relevance in all groups, even with the largest number of missing values, as shown in Fig 3a–3d. However, there is a decrease in the weights of the PL variable with the increase in the percentage of missing values in Cluster 2 for the PDS and OCS methods. This behavior is also observed for the weights of the PW variable in Cluster 1 in the PDS method. For the WDS strategy, as the percentage of missing values increases, variable PW becomes more relevant.

**Table 3:** Weights of the variables in each group adjusted by the VKFCM-K-LP algorithm with the WDS strategy under different percentages of missing values.

% NA	Cluster	Weights			
		SL	SW	PL	PW
**5**	1	0.5037	0.1256	**4.9758**	**3.1759**
	2	0.6373	0.4769	**2.2666**	**1.4512**
	3	0.5558	0.5889	**2.3945**	**1.2758**
**10**	1	0.4921	0.1092	**5.3030**	**3.5064**
	2	0.5829	0.4588	**2.3282**	**1.6057**
	3	0.6278	0.6350	**2.2436**	**1.1177**
**15**	1	0.5193	0.1112	**4.9059**	**3.5269**
	2	0.6167	0.4545	**2.0929**	**1.7041**
	3	0.5142	0.6845	**2.0667**	**1.3744**
**20**	1	0.4840	0.0961	**4.7588**	**4.5156**
	2	0.5645	0.4023	**2.3154**	**1.9013**
	3	0.5618	0.6328	**2.4836**	**1.1322**

**Table 4:** Weights of the variables in each group adjusted by the VKFCM-K-LP algorithm with the PDS strategy under different percentages of missing values.

% NA	Cluster	Weights			
		SL	SW	PL	PW
**5**	1	0.4825	0.1349	**5.1574**	**2.9772**
	2	0.5606	0.5797	**2.4196**	**1.2713**
	3	0.6293	0.4658	**2.2921**	**1.4879**
**10**	1	0.4753	0.1317	**5.3963**	**2.9595**
	2	0.6459	0.4525	**2.2600**	**1.5135**
	3	0.5799	0.6772	**2.2523**	**1.1304**
**15**	1	0.5011	0.1345	**5.1671**	**2.8709**
	2	0.7530	0.4172	**2.2037**	**1.4443**
	3	0.5390	0.8078	**2.1712**	**1.0575**
**20**	1	0.5011	0.1345	**5.1671**	**2.8709**
	2	0.7530	0.4172	**2.2037**	**1.4443**
	3	0.5390	0.8078	**2.1712**	**1.0575**

**Table 5:** Weights of the variables in each group adjusted by the VKFCM-K-LP algorithm with the OCS strategy under different percentages of missing values.

% NA	Cluster	Weights			
		SL	SW	PL	PW
**5**	1	0.4876	0.1363	**5.1732**	**2.9078**
	2	0.6364	0.4663	**2.2082**	**1.5256**
	3	0.5590	0.5811	**2.4149**	**1.2746**
**10**	1	0.4744	0.1317	**5.3949**	**2.9642**
	2	0.6447	0.4468	**2.1660**	**1.6024**
	3	0.5545	0.6754	**2.2918**	**1.1648**
**15**	1	0.5062	0.1338	**5.1193**	**2.8821**
	2	0.7345	0.4044	**2.1258**	**1.5835**
	3	0.5219	0.7826	**2.2277**	**1.0989**
**20**	1	0.4576	0.1210	**5.4762**	**3.2964**
	2	0.7185	0.4041	**2.0228**	**1.7024**
	3	0.5122	0.7171	**2.4697**	**1.1020**

**Fig 4:**
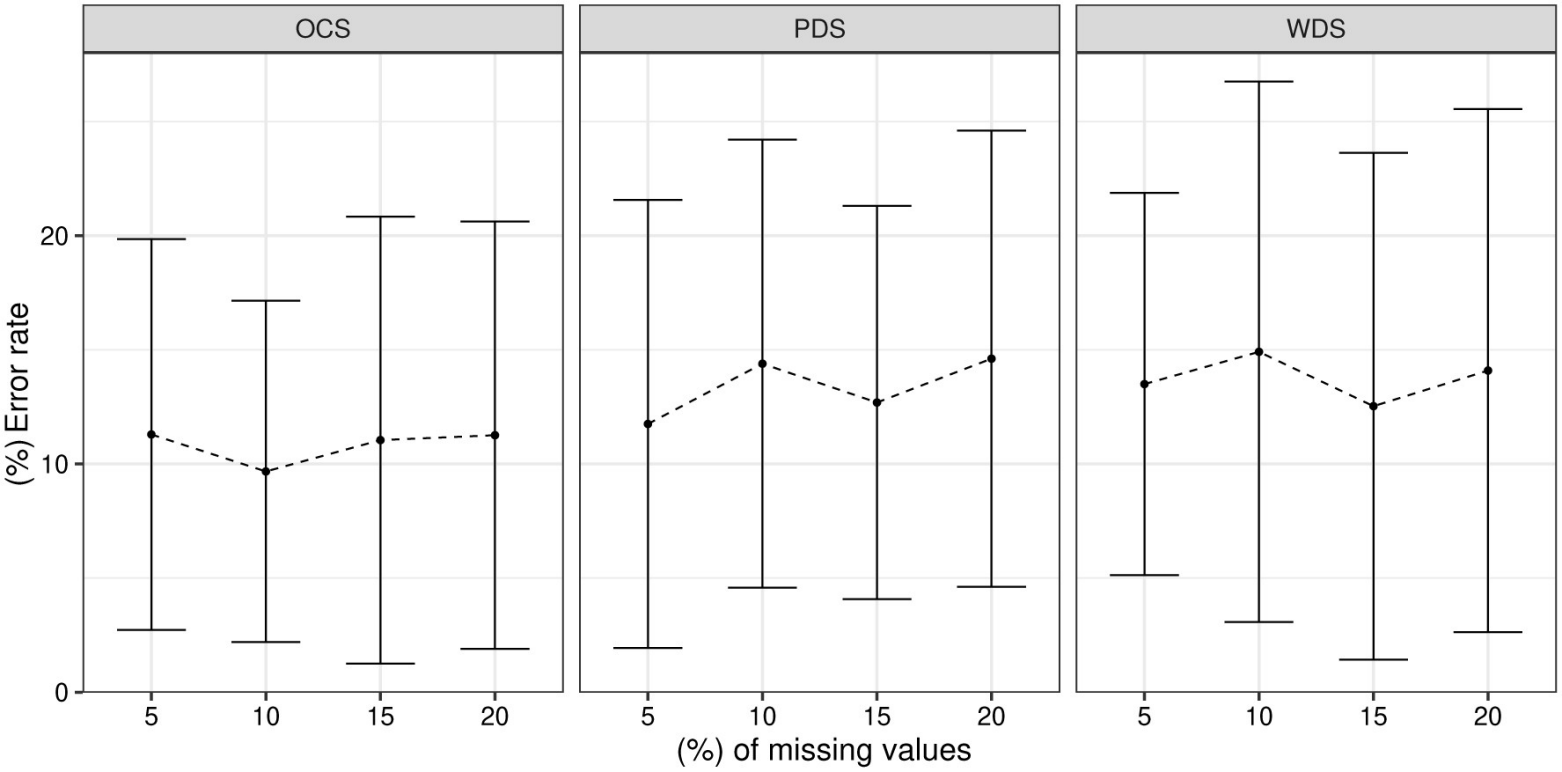
Average error rates after 100 repetitions for the *Iris Plant* dataset.

Fig 4 shows the performance results of the OCS, PDS and WDS algorithms in the 100 Monte Carlo repetitions. The WDS strategy had the largest deviations in error rate when compared to the others. For the PDS approach, increasing and decreasing average error rates were observed over the analyzed percentages. In the OCS strategy, there is an increasing error rate, starting from 10% of missing values. This method presents a more defined behavior, i.e. as the percentage of missing values increases, the error rate also increases. The OCS strategy showed the smallest deviations in relation to the average error rate when compared with the WDS and PDS strategies.

Analyzing the measures of variable consistency from Table 6, considering the complete dataset obtained after clustering with the VKFCM-K-LP algorithm, together with the OCS strategy, we have that these measures are very close to zero. This shows a good quality in the grouping, that is, the new values imputed through the OCS strategy were not discrepant in relation to the original scale of the variables of the *Iris Plant* dataset.

**Table 6:** Consistency of variables for the dataset *Iris Plant*.

% NA	Cluster	Weights			
		SL	SW	PL	PW
**5**	1	0.00025	0.00115	0.00534	0.00067
	2	0.00355	0.00035	0.00845	0.06201
	3	0.00583	0.00271	0.01433	0.00000
**10**	1	0.00040	0.00485	0.00563	0.00001
	2	0.00785	0.00154	0.01366	0.06181
	3	0.00952	0.01112	0.03800	0.00295
**15**	1	0.00012	0.01161	0.01162	0.00029
	2	0.00537	0.00181	0.06160	0.06618
	3	0.01623	0.02967	0.03063	0.00266
**20**	1	0.00673	0.02212	0.02356	0.00263
	2	0.01598	0.00622	0.10373	0.12329
	3	0.02470	0.03045	0.01900	0.11264

### 8.2 *Thyroid Gland* dataset

In this Section, we evaluate the three missing data approaches using the *Thyroid Gland* dataset [67]. This dataset has three *a priori* classes (*K* = 3): normal (Class 1) with 150 observations, hyper (Class 2), with 35 observations and hypo (Class 3) with 30 observations. This dataset has *n* = 215 observations and five variables (*p* = 5): T3-resin uptake test (T3), Total Serum thyroxin (TTS), Total serum triiodothyronine (TST), basal thyroid-stimulating hormone (TSH) and Maximal absolute difference of TSH value after injection of 200 micrograms of thyrotropin-releasing hormone (DTSH).

**Fig 5:**
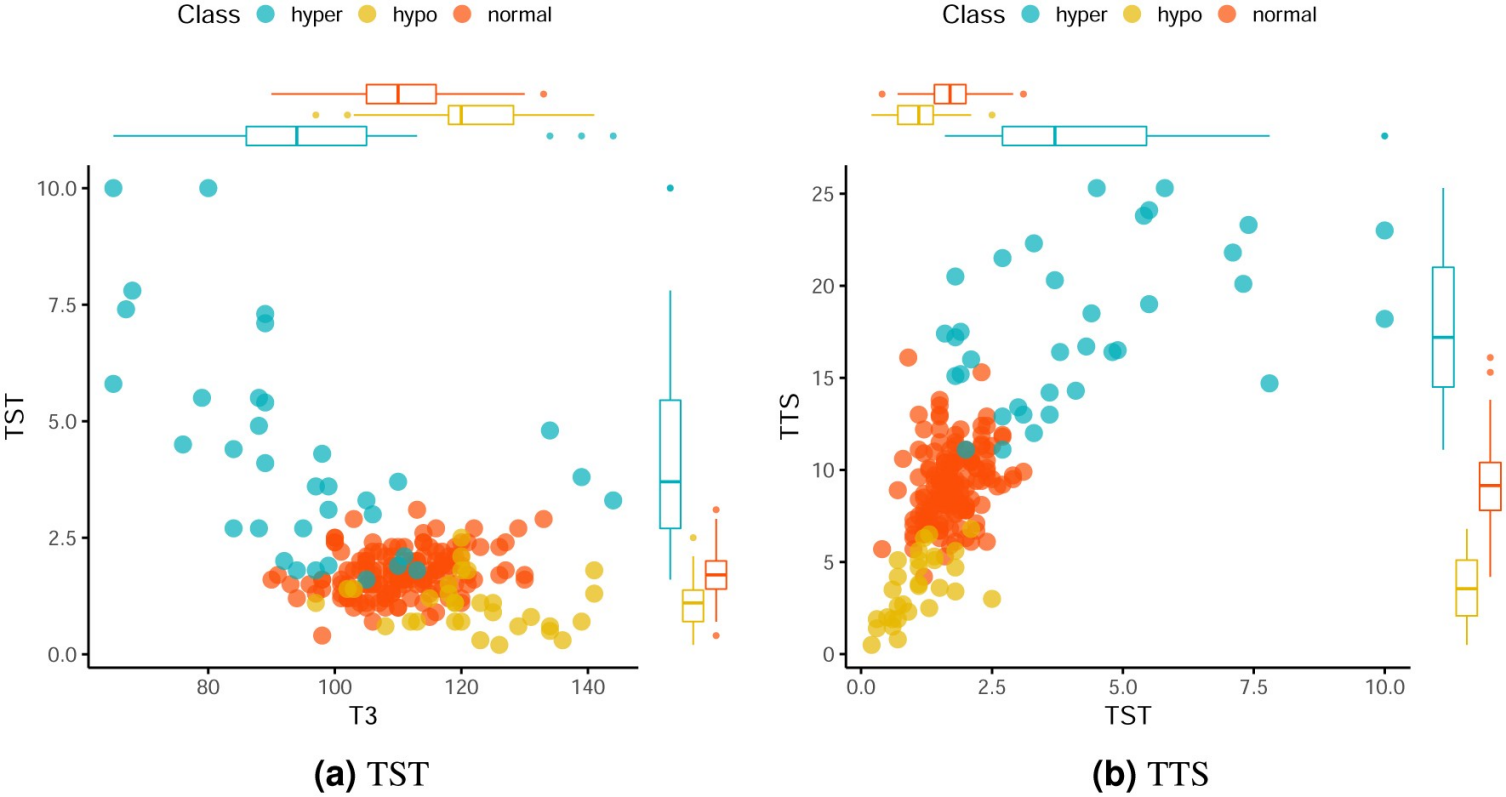
Scatter plots and boxplots for the *Thyroid Gland* dataset. (**a**) TST. (**b**) TTS.

Fig 5a and 5b present the dispersion and boxplot graphs for T3 plotted against TST and TST versus TTS. Class 2 is more dispersed than the others, which is evidenced in the boxplots for the analyzed variables. Fig 5b shows a linear relationship between variables TST and TTS for classes 1 and 2. In addition, these classes have less variability when considering the TST variable.

**Fig 6:**
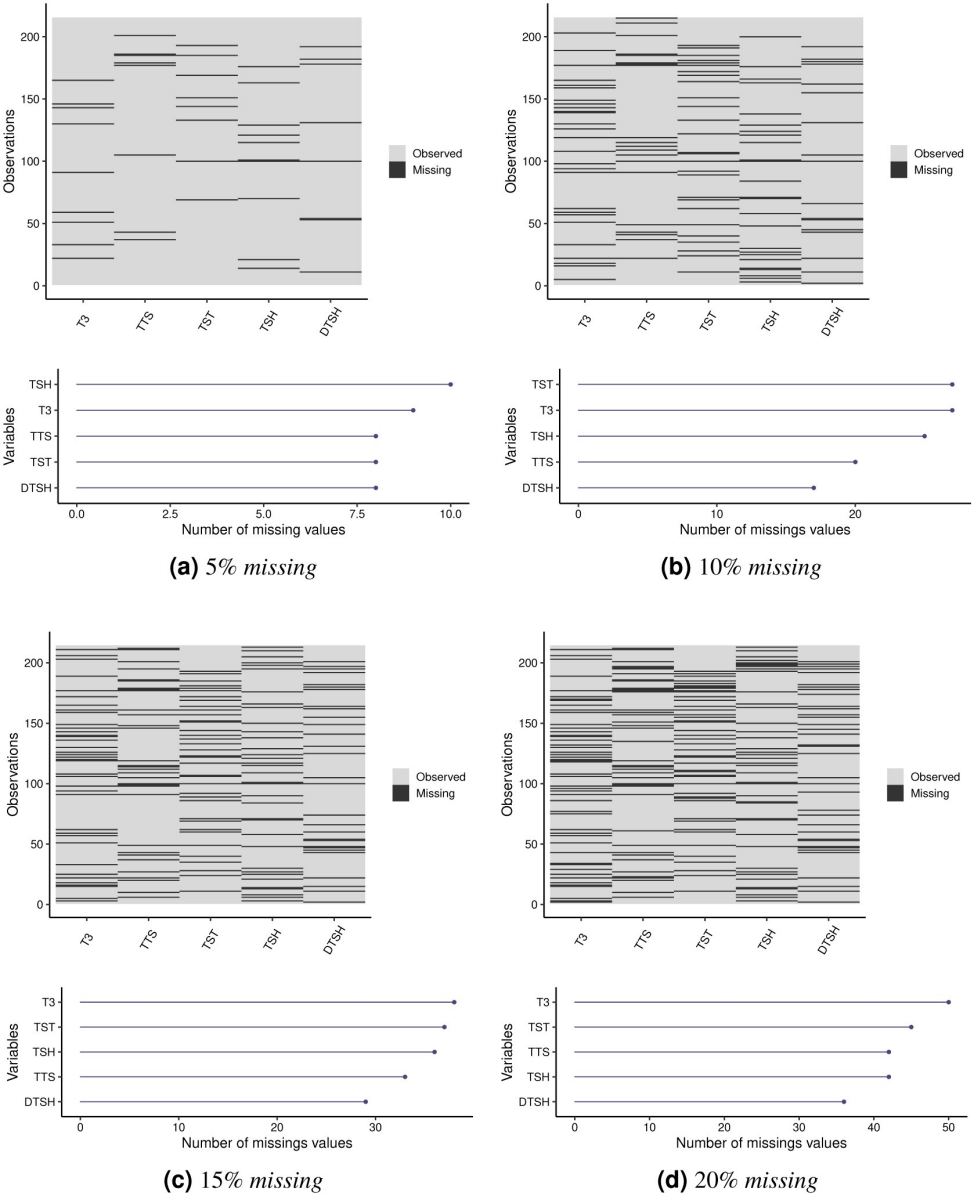
Graphs of missing value patterns and frequencies per variable for the *Thyroid Gland* dataset. (**a**) 5% *missing*. (**b**) 10% *missing*. (**c**) 15% *missing*. (**d**) 20% *missing*.

Fig 6a–6d show the missing values distributed across the five variables in the *Thyroid Gland* dataset. Variable T3 presents a greater number of missing values for the 15% and 20% percentages. For all analyzed datasets, the DTSH variable has the smallest amount of missing values. Additionally, the missing values are well distributed among the variables. Observations in the 1|–150 range represent class 1 (normal), the 151|–175 interval corresponds to class 2 (hyper) and finally, interval 175|–215 contains class 3 (hypo) observations.

Table 7 shows the best results among the 100 repetitions of the VKFCM-K-LP algorithm under the three types of strategies for missing data. For 5% of missing values, the best performances were obtained by the WDS method, presenting a CR equal to 0.818 and an FM equal to 0.943, which means there was a good agreement between the *a priori* classes and the clusters provided by the clustering algorithm. In this context, the OERC measure was equal to 5.5%. For the PDS and OCS strategies, the increase in the number of missing values in the *Thyroid Gland* dataset influences the quality of the clustering, as there was a decrease in the values of the studied measures. The PDS strategy showed the best performances according to the quality measures analyzed for all percentages of missing values.

**Table 7:** Performance of the VKFCM-K-LP clustering algorithm with the WDS, PDS and OCS strategies for the *Thyroid Gland* dataset.

% NA	CR			FM			OERC		
	EDC	EDP	ECO	EDC	EDP	ECO	EDC	EDP	ECO
**5**	0.818	0.803	0.775	0.943	0.939	0.930	0.055	0.060	0.069
**10**	0.509	0.734	0.656	0.838	0.918	0.892	0.176	0.083	0.111
**15**	0.787	0.633	0.586	0.935	0.885	0.868	0.065	0.120	0.139
**20**	0.753	0.441	0.434	0.923	0.809	0.807	0.074	0.204	0.200

To build the confusion matrices in Table 8, the clusters provided by the algorithm were identified as Cluster 1 (normal), Cluster 2 (hyper) and Cluster 3 (hypo). The confusion matrices show a great difficulty for the clustering algorithm in identifying Clusters 1 and 3 in all the methods analyzed. These clusters correspond to the normal and hypo classes, which in Fig 5a and 5b are more overlapped when compared to Class 2, which hinders the performance of the clustering method.

**Table 8:** Confusion matrices obtained by VKFCM-K-LP with the WDS, PSD and OCS strategies using 5, 10, 15 and 20% of missing values.

Methods	Clusters	5%			10%			15%			20%		
		1	2	3	1	2	3	1	2	3	1	2	3
WDS	1	144	0	6	118	1	4	143	2	5	147	5	8
	2	6	35	0	32	34	1	7	33	0	3	30	0
	3	0	0	24	0	0	25	0	0	25	0	0	22
PDS	1	143	0	6	138	1	5	130	1	5	117	2	8
	2	7	35	0	12	34	0	20	34	0	33	33	1
	3	0	0	24	0	0	25	0	0	25	0	0	21
OCS	1	141	0	6	133	1	6	127	1	6	115	2	7
	2	9	35	0	17	34	0	23	34	0	35	33	1
	3	0	0	24	0	0	24	0	0	24	0	0	22

**Fig 7:**
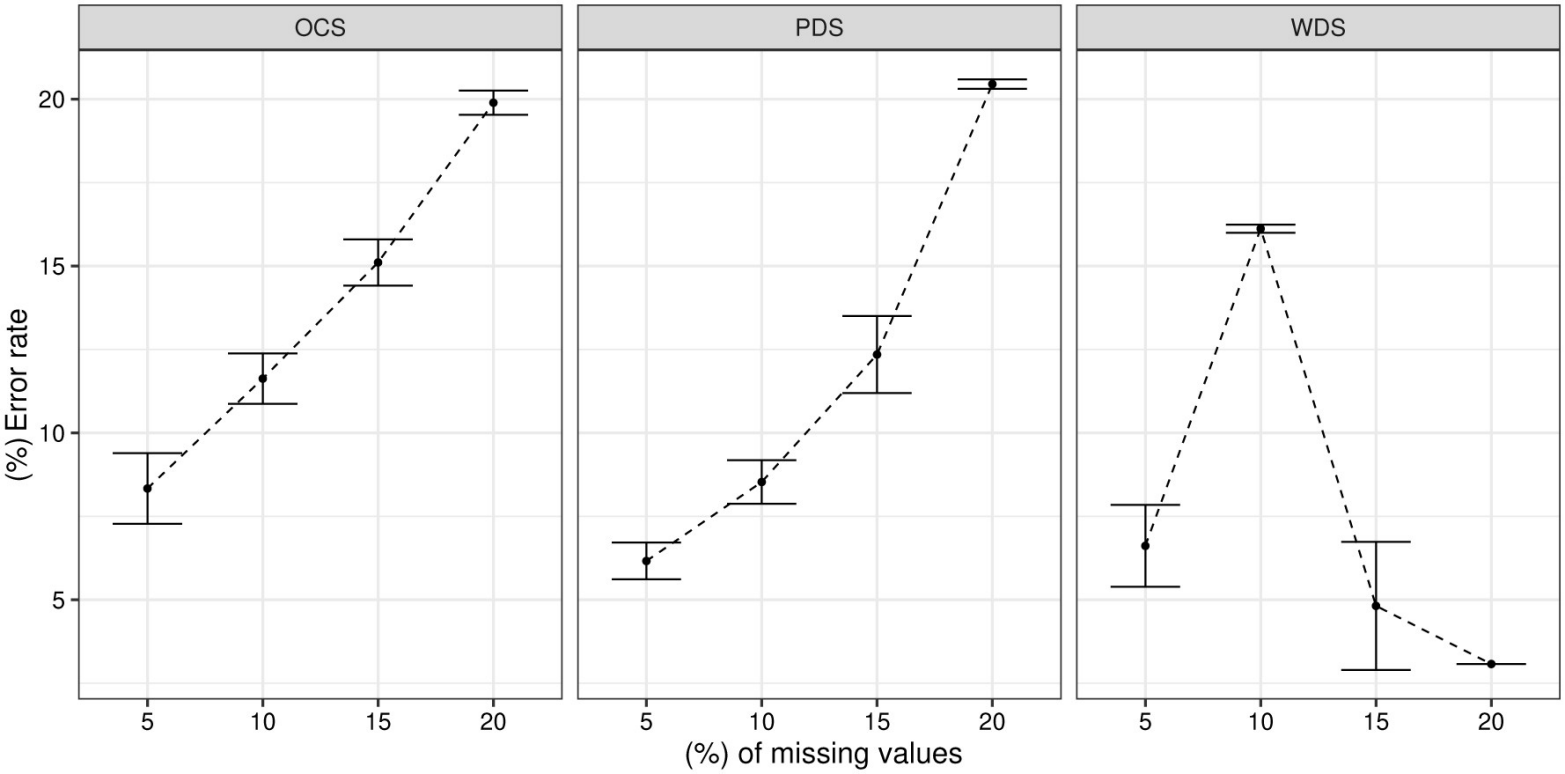
Average results of 100 repetitions for the error rate with *Thyroid Gland* dataset.

Fig 7 presents the average error rates for the 100 repetitions of the VKFCM-K-LP algorithm, with the WDS, PDS and OCS strategies in the *Thyroid Gland* dataset. The average error rates for PDS and OCS showed an increasing behavior along the percentages of missing values evaluated. For 20% of missing values, the average Total Error Rate of classification for these methods was approximately 0.20.

The PDS method presents lower error rates from 5% to 15% of missing values, when compared to the OCS strategy. The largest variations are observed in the WDS method for 5% and 15% of missing values. This method obtained an increasing error rate between 5% and 10%, while its error decreased starting from 10%.

The weights of the variables in each cluster, with the WDS, PDS and OCS approaches, listed in Tables 9–11, show that the TST and TSH variables were the most relevant to compose Cluster 1. For Cluster 2, the most important variables were TSH and STD and, for Cluster 3, the most relevant variables were TTS and TST. In addition, in the WDS strategy the TTS and TSH variables were more relevant for the construction of Clusters 3 and 2 respectively, as the number of missing values increased. This behavior is also observed for the TST variable in Cluster 1 for the PDS and OCS strategies. In contrast, with the increase in the number of missing values in the DTSH variable, there was a decrease in its importance for the construction of Cluster 2 with the OCS strategy.

**Table 9:** Weights of the variables in each group found by the VKFCM-K-LP algorithm with the WDS strategy under different percentages of missing values.

% NA		Weights				
		T3	TTS	TST	TSH	DTSH
**5**	1	0.4469	0.6842	**0.9406**	**5.3455**	0.6502
	2	0.2463	0.2615	0.1708	**15.3753**	**5.9078**
	3	1.3989	**3.2601**	**3.1756**	0.2943	0.2345
**10**	1	0.5495	0.7670	**1.1412**	**2.9349**	0.7082
	2	0.2504	0.2753	0.1993	**17.6822**	**4.1143**
	3	1.8367	**3.3095**	**3.0556**	0.2484	0.2166
**15**	1	0.4591	0.7436	**1.1079**	**3.9960**	0.6614
	2	0.2034	0.2707	0.1294	**19.3835**	**7.2343**
	3	1.3438	**3.9067**	**3.1396**	0.2588	0.2343
**20**	1	0.4863	0.7701	1.2057	3.9571	0.5595
	2	0.1909	0.2899	0.1010	**21.5217**	**8.3067**
	3	1.1509	**4.3158**	**3.4958**	0.2505	0.2298

**Table 10:** Weights of the variables in each group found by the VKFCM-K-LP algorithm with the PDS strategy under different percentages of missing values.

% NA		Weights				
		T3	TTS	TST	TSH	DTSH
**5**	1	0.4379	0.6721	**0.9378**	**5.5693**	0.6503
	2	0.2197	0.2613	0.1653	**16.3745**	**6.4314**
	3	1.3780	**3.4215**	**3.2403**	0.2928	0.2234
**10**	1	0.4666	0.6584	**0.9903**	**5.1460**	0.6385
	2	0.2303	0.2467	0.1711	**15.662**	**6.5622**
	3	1.4038	**3.3756**	**3.5690**	0.2763	0.2139
**15**	1	0.4792	0.6781	**1.0123**	**5.2929**	0.5742
	2	0.2548	0.2261	0.1784	**16.4694**	**5.9023**
	3	1.2320	**3.6878**	**3.5524**	0.2785	0.2224
**20**	1	0.4973	0.6409	**1.0404**	**6.0578**	0.4976
	2	0.3227	0.2245	0.2006	**15.8473**	**4.3391**
	3	1.1033	**4.8855**	**4.0218**	0.2199	0.2097

**Table 11:** Weights of the variables in each group found by the VKFCM-K-LP algorithm with the OCS strategy under different percentages of missing values.

% NA		Weights				
		T3	TTS	TST	TSH	DTSH
**5**	1	0.4403	0.6634	**0.9334**	**5.5916**	0.6557
	2	0.2187	0.2711	0.1691	**16.4355**	**6.0643**
	3	1.3729	**3.4176**	**3.2644**	0.2926	0.2230
**10**	1	0.4888	0.6737	**0.9699**	**5.0058**	0.6253
	2	0.2361	0.2653	0.1863	**16.7371**	**5.1163**
	3	1.3996	**3.3082**	**3.6077**	0.2827	0.2117
**15**	1	0.4979	0.7017	**1.0009**	**4.6589**	0.6137
	2	0.2656	0.2554	0.2031	**17.3437**	**4.1813**
	3	1.2947	**3.4309**	**3.2439**	0.3200	0.2168
**20**	1	0.4921	0.6576	**1.0223**	**5.1672**	0.5848
	2	0.3335	0.2552	0.2292	**16.5093**	**3.1038**
	3	1.1178	**4.3838**	**3.4333**	0.2940	0.2021

In order to assess the consistency of the variables in each cluster, as shown in Table 12, we used the datasets before and after clustering, with the missing values imputed using the OCS strategy. In this context, the consistencies obtained for the variables in the groups were close to zero, which indicates a good performance of the OCS method when imputing the missing values. Additionally, the greatest consistencies were found in Clusters 2 and 3 for all percentages of missing values evaluated.

**Table 12:** Consistencies of variables for the *Thyroid Gland* dataset.

% NA		Weights				
		T3	TTS	TST	TSH	DTSH
**5**	1	0.01258	0.00092	0.00030	0.00238	0.00529
	2	0.00407	0.04769	0.04562	0.00813	0.06072
	3	0.00000	0.00691	0.00414	0.00000	0.01321
**10**	1	0.01514	0.03953	0.02637	0.00196	0.00063
	2	0.02250	0.09938	0.14733	0.01706	0.22179
	3	0.01783	0.12019	0.00961	0.00378	0.01297
**15**	1	0.02515	0.02872	0.01589	0.00372	0.01526
	2	0.02845	0.16012	0.22214	0.01981	0.27982
	3	0.03377	0.13482	0.00927	0.06684	0.05777
**20**	1	0.04188	0.03056	0.02345	0.00493	0.00116
	2	0.04946	0.28002	0.34943	0.02413	0.40891
	3	0.03002	0.20914	0.02891	0.11276	0.08566

### 8.3 Comparison between imputation methods

**Fig 8:**
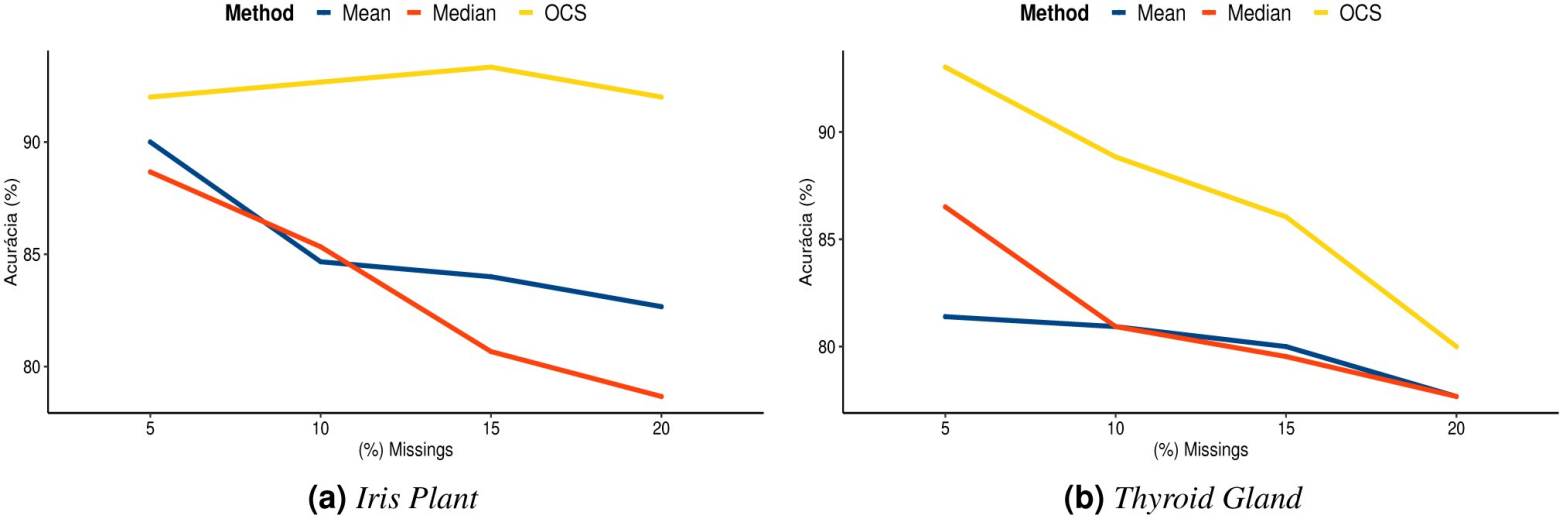
Performance graphs of the methods for different percentages of missing values. (**a**) *Iris Plant*. (**b**) *Thyroid Gland*.

This Section compares VKFCM-K-LP using the OCS method with *Imputation via Mean and Median*. Fig 8a and 8b show the accuracies obtained using the OCS method and by Imputation via Mean and Median for the *Iris Plant* and *Thyroid Gland* datasets, when the amount of missing values varies from 5 to 20%.

For the *Imputation via Mean and Median* values, missing values are filled using the mean or median estimates of the observed values in the datasets for each variable, before applying the clustering algorithm.

**Fig 9:**
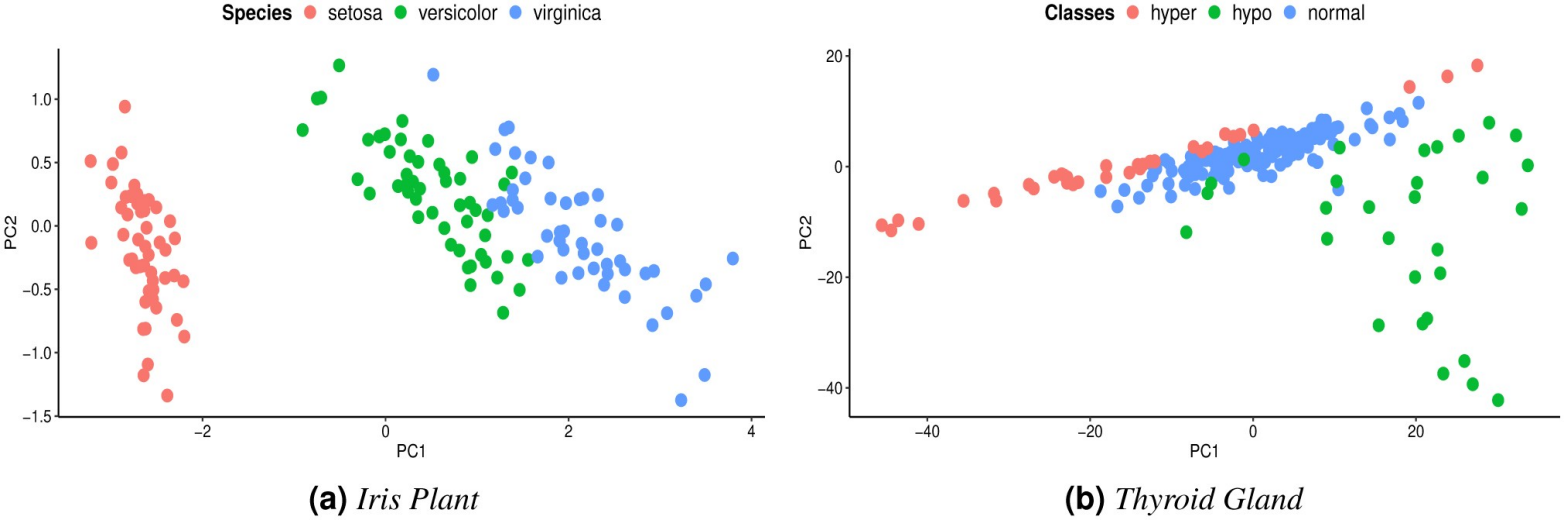
Principal component analysis applied to both datasets. (**a**) *Iris Plant*. (**b**) *Thyroid Gland*.

For the *Iris Plant* dataset with 5% of missing values, accuracy was close to 0.90, which shows a good performance of the methods when imputing missing values. However, for the *Thyroid Gland* dataset, considering the same percentage of missing values, there are differences in accuracy as shown in Fig 8a. This difference between the two datasets is expected, because the classes in the *Thyroid Gland* dataset are more overlapped than the classes in the *Iris Plant* dataset. In order to visualize and understand the data overlap, we applied Principal Component Analysis (PCA). Fig 9a and 9b show the resulting projections for the first two components. In PCA, the components are orthogonal and sorted according to how much variance they explain, so it is possible to identify patterns and extract features [69]. Even after applying PCA, the classes in the *Thyroid Gland* dataset are more overlapped than the classes in the *Iris Plant* dataset. This makes it harder to group observations in the *Thyroid Gland* dataset. This difficulty is accentuated with the increase in the number of missing values in the dataset as shown in Fig 8b. It is also worth mentioning that classes overlap less in the *Iris Plant* dataset, which favors the performance of the VKFCM-K-LP clustering method with the OCS strategy, even when the percentage of missing values increases, as shown in Fig 8b.

For 10%, 15% and 20% of imputed missing values, the clustering accuracies with the imputation by Mean and Median values in the *Thyroid Gland* dataset are concentrated around very similar values, which does not happen in the *Iris Plant* dataset (Fig 8a).

Tables 13–16 show the consistencies of the variables with the imputation of the missing values via Mean and Median for the two analyzed datasets. Consistencies obtained by the Mean and Median strategies were higher than the OCS strategy, as shown in Tables 6–12. This means that Mean and Median imputations depart more from the original scale of the variables in the two datasets than values obtained by the OCS approach.

**Table 13:** Consistency of variables in the VKFCM-K-LP clustering with the imputation of missing values using mean values for the *Iris Plant* dataset.

% NA	Cluster	Weights			
		SL	SW	PL	PW
**5**	1	0.01731	0.01634	0.17422	0.25311
	2	0.00890	0.05284	0.03431	0.05032
	3	0.02456	0.00624	0.21387	0.00000
**10**	1	0.05468	0.05008	0.17244	0.32902
	2	0.01209	0.07793	0.08767	0.06302
	3	0.12788	0.00598	0.38896	0.09438
**15**	1	0.18425	0.12677	0.41515	0.43382
	2	0.07385	0.10341	0.15307	0.03371
	3	0.12372	0.02019	0.41156	0.09245
**20**	1	0.17918	0.12613	0.53173	0.49124
	2	0.10224	0.11974	0.18223	0.03556
	3	0.19865	0.01895	0.50715	0.23976

**Table 14:** Consistency of variables in the VKFCM-K-LP clustering with the imputation of missing values using median values for the *Iris Plant* dataset.

% NA	Cluster	Weights			
		SL	SW	PL	PW
**5**	1	0.01575	0.01845	0.18338	0.25776
	2	0.00551	0.04521	0.02264	0.02167
	3	0.02632	0.00879	0.16321	0.00000
**10**	1	0.04675	0.05812	0.18284	0.33365
	2	0.03029	0.06797	0.03022	0.02191
	3	0.14485	0.00464	0.30564	0.08778
**15**	1	0.17198	0.13849	0.42178	0.44085
	2	0.09633	0.09032	0.04010	0.05905
	3	0.13630	0.04332	0.32237	0.07927
**20**	1	0.16297	0.13849	0.53969	0.49929
	2	0.13870	0.10174	0.02639	0.10506
	3	0.21722	0.04332	0.40975	0.19766

**Table 15:** Consistency of variables in the VKFCM-K-LP clustering with the imputation of missing values using mean values for the *Thyroid Gland* dataset.

% NA		Weights				
		T3	TTS	TST	TSH	DTSH
**5**	1	0.00891	0.00020	0.01779	0.09343	0.03028
	2	0.02228	0.14048	0.02442	0.17675	0.23741
	3	0.00000	0.17499	0.02278	0.00000	0.00214
**10**	1	0.05014	0.00643	0.04407	0.25214	0.05116
	2	0.13232	0.16339	0.08427	0.24967	0.39328
	3	0.02532	0.23810	0.03123	0.01299	0.00230
**15**	1	0.03076	0.01280	0.08501	0.25876	0.08239
	2	0.16093	0.20753	0.12637	0.23315	0.43141
	3	0.07188	0.36764	0.03087	0.16200	0.03564
**20**	1	0.03848	0.01942	0.08920	0.25748	0.10013
	2	0.22618	0.32794	0.17708	0.22526	0.51254
	3	0.06937	0.44036	0.00793	0.21541	0.11537

**Table 16:** Consistency of variables in the VKFCM-K-LP clustering with the imputation of missing values using median values for the *Thyroid Gland* dataset.

% NA		Weights				
		T3	TTS	TST	TSH	DTSH
**5**	1	0.01004	0.00392	0.00398	0.02499	0.00407
	2	0.02254	0.14674	0.03345	0.05759	0.21036
	3	0.00000	0.16661	0.00844	0.00000	0.00121
**10**	1	0.05661	0.02413	0.01215	0.02623	0.00971
	2	0.13441	0.17277	0.10566	0.08908	0.36343
	3	0.02321	0.22150	0.00423	0.01625	0.00121
**15**	1	0.04232	0.01905	0.00615	0.03655	0.02563
	2	0.16424	0.22067	0.15439	0.08908	0.39172
	3	0.06671	0.34919	0.00423	0.18079	0.04952
**20**	1	0.04668	0.01045	0.00728	0.03869	0.01671
	2	0.22855	0.34195	0.21072	0.08908	0.47284
	3	0.06671	0.41992	0.03112	0.24134	0.13076

**Fig 10:**
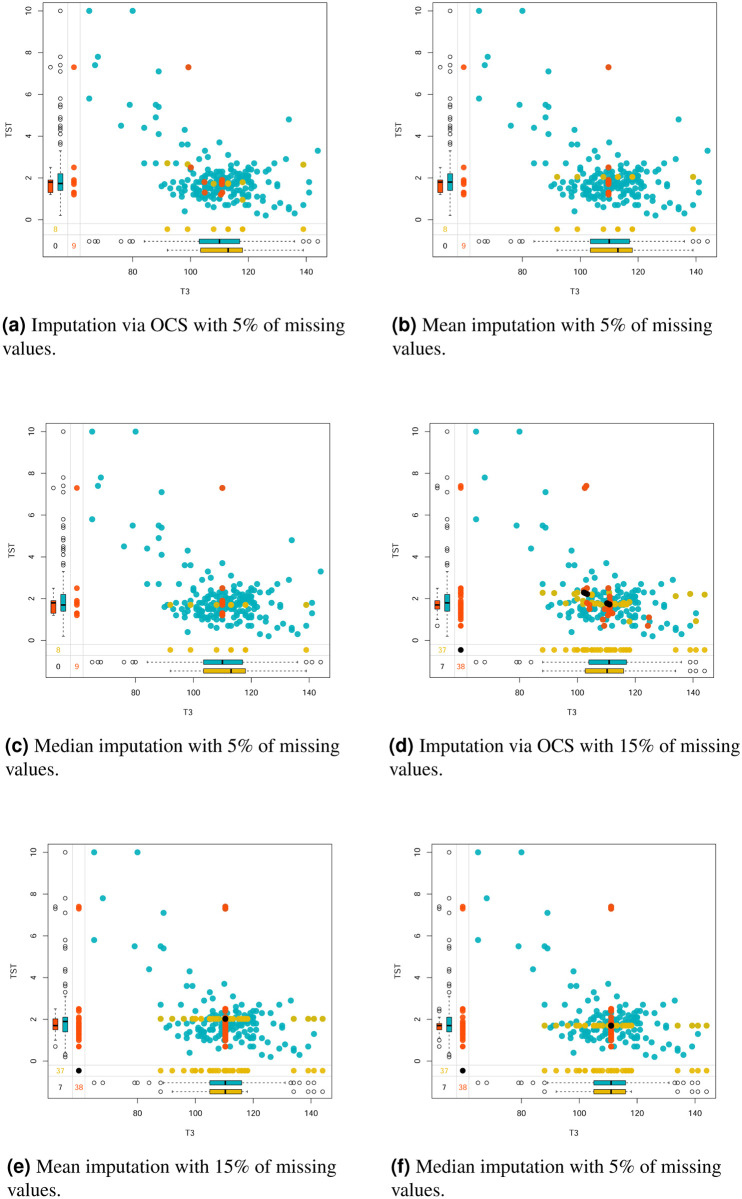
Scatter plots and boxplots for the *Thyroid Gland* dataset considering the different imputation methods. (**a**) Imputation via OCS with 5% of missing values. (**b**) Mean imputation with 5% of missing values. (**c**) Median imputation with 5% of missing values. (**d**) Imputation via OCS with 15% of missing values. (**e**) Mean imputation with 15% of missing values. (**f**) Median imputation with 5% of missing values.

To show the dispersion of the new values imputed using the strategies mentioned above, variables T3 and TST were selected from the *Thyroid Gland* dataset with 5% and 15% of missing values. The T3 and TST variables were those that obtained the highest number of missing values in the generation process (see Fig 6b and 6c). Therefore, it is important to graphically visualize the relationship of these imputed values with the ones in the dataset, as shown in Fig 10a–10f.

The box-plots for the imputed values and the complete values (blue color) are also shown. The red and yellow colors represent imputed values for the T3 and TST variables respectively. If the values are imputed to both variables, they are colored black. Fig 10a–10c show that, with 5% of imputed missing values, most of the resulting points are close to the distribution of the complete data. The boxplots of the imputed values for the TST variable show higher similarity with the boxplots of the complete values, that is, the dispersion of the data before and after the imputation did not present significant discrepancies.

Regarding the T3 variable, the imputed values showed less variability than the complete observations. For datasets with 15% of imputed values (Fig 10d–10f), the values obtained by the OCS strategy showed better distribution in the groups compared to the values imputed with Mean and Median estimates.

In the imputation of missing values via Mean and Median, there is a concentration in the same value, forming a straight line with zero slope. Thus the set of values imputed through these methods has zero correlation between variables T3 and TST. Tabachnick et al. [70] argue that the imputation of missing values with central tendency measures such as the average, affects the correlation between the variables and the variance is underestimated.

Indeed when analyzing the correlations of variables T3 and TST, we obtain *ρ* = −0.528 and *ρ* = −0.529, after imputation with Mean and Median, respectively. Meanwhile, the correlation for the original set (without missing values) is *ρ* = −0.536. The variability of the data is also impaired, as the standard deviations (*sd*) for the T3 and TST variables in the complete dataset were *sd* = 13, 145 and *sd* = 1, 419, respectively, while for the set with 15% of imputed values, the standard deviations are *sd* = 11.87 and *sd* = 1.35 respectively, which indicates variance underestimation.

Therefore, although the imputations using Mean and Median values are easy to implement, the resulting clusterings are not satisfactory, since the structure of the correlation of the variables is modified and consequently these new values may not be related to their group of origin, as shown in Fig 10b, 10e and 10f. Finally, the VKFCM-K-LP method with the OCS strategy showed better performance in identifying *a priori* classes, according to the accuracies observed in Fig 8a and 8b, so the set of values imputed using this strategy is closer to the set of observations from the original dataset shown in Fig 5a.

## 9 Conclusions

The problem of missing data is commonly discussed in several areas of science, as statistical techniques used for data analysis, such as clustering, were originally proposed for datasets without missing values. An alternative to face this issue is to adapt the clustering methods so that they can handle incomplete datasets. In this work, the VKFCM-K-LP clustering method was studied with three types of strategies to deal with missing data, WDS, PDS and OCS. In order to evaluate clustering methods in the context of missing data, two benchmark datasets were used: *Iris Plant* and *Thyroid Gland*.

From these datasets, new datasets with 5%, 10%, 15% and 20% of missing values were artificially generated. The results of the clustering algorithms were evaluated according to CR, FM and OERC. The results of the clustering for the *Iris Plant* dataset were satisfactory, with CR and FM close to 1 and the OERC measure close to zero, for all analyzed methods and percentages of missing values, which showed a good performance of the VKFCM-K-LP method under the WDS, PDS and OCS approaches in identifying *a priori* classes. For 5% of missing values the best performance of the VKFCM-K-LP clustering algorithm was observed with the PDS strategy. However, the performance graph for the 100 repetitions of the algorithm shows that for 10%, 15% and 20% of missing values, this method had the poorest performance. Additionally, the confusion matrices showed that observations belonging to Class 1 (setosa) in the *Iris Plant* dataset were properly grouped.

Regarding the weights of the variables in each group, variable PL was the most relevant, even with a higher percentage of missing values. The measures of consistency of the variables for the datasets obtained from the grouping with the VKFCM-K-LP algorithm, together with the OCS strategy, were close to zero, which showed a good clustering quality, that is, the values imputed using the OCS method were not discrepant in relation to the original scale of the variables.

In the generation of missing values for the *Thyroid Gland* dataset, variable T3 presented a greater amount of these values for 15% and 20% of missing values. The best quality measures for this dataset were observed in the PDS method. In addition, the methods showed an increasing average error rate when analyzing the performance graph on the 100 repetitions of the algorithm. The confusion matrices for the *Thyroid Gland* dataset showed an overlap between Classes 1 and 2 in all methods analyzed, which corresponded to a greater number of incorrectly grouped observations when compared with Class 3.

Variables TSH and DTSH obtained the highest weights in the construction of Cluster 2 in all analyzed cases. In contrast, variable T3 had little influence on the formation of the groups. The consistencies of the variables obtained for the OCS method in the *Thyroid Gland* dataset were close to zero, which means a good performance of the method in imputing the missing values.

When comparing the clustering results using the OCS and the Average and Median imputation methods, we have found that the best accuracy was observed for the OCS method in all considered percentages of missing values for both analyzed datasets. The results of the VKFCM-K-LP clustering using the imputation methods with the Mean and Median did not present satisfactory results, because the set of imputed values affected the general correlation of the variables in the dataset and there was a distortion in the variability of the data, which affected the quality of the clusters.

In general, the VKFCM-K-LP clustering algorithm together with the missing data strategies WDS, PDS and OCS presented satisfactory results in the datasets with 5% 10%, 15% and 20% of missing values. The best performances obtained by the grouping method were observed when paired with the PDS and OCS strategies. In the groups made with the OCS approach, new datasets were derived and the missing values were estimated in the optimization process. The results of the clustering with the OCS strategy showed superior performances when compared to the results obtained by imputing with the mean and median of the observed values.
